# Effect of blending ratio of wheat, orange fleshed sweet potato *(Ipomoea batatas L*.) powder and haricot bean ( *Phaseolus vulgaris* *L*.) flour on proximate composition, physical properties and sensory acceptability of biscuits

**DOI:** 10.12688/f1000research.52634.1

**Published:** 2021-06-28

**Authors:** Fieben Kindeya, Welday Hailu, Tilku Dessalegn, Gesessew L. Kibr

**Affiliations:** 1 Department of Food and Nutritional Sciences, Shambu Campus, Wollega University, Shambu Town, Oromia Region, 38, Ethiopia; 2 School of Nutrition, Food Science and Technology, Hawassa University, Hawassa, Sidama Region, Ethiopia

**Keywords:** Protein-Energy Malnutrition; Biscuit; Haricot bean; Sensory acceptability

## Abstract

Background

Protein-energy deficiency (PEM) is still a major health issue in developing countries, and it is the leading cause of disease and death in children under the age of 5 years.

Methods

100:0:0; 90:5:5; 80:10:10; 70:15:15; 60:20:20; and 50:25:25 per cent wheat:haricot bean: orange-fleshed sweet potato (OFSP) flours were used to make composite cookies. Standard methods were used to evaluate the proximate structure, physical properties, and sensory assessment. A one-way analysis of variance (ANOVA) model was used to statistically evaluate the data using the statistical analysis system (SAS) software package, version 9.0 standard methods.

Results

The results showed that partially replacing wheat with haricot bean and OFSP increased the proximate composition significantly. When wheat was replaced with haricot bean and OFSP, the physical characteristics of the biscuits did not vary significantly from those of biscuits made entirely of wheat flour. Sensory acceptability (appearance, color, flavor, taste, and overall acceptability) was higher in the composite biscuits with up to 40% wheat substitution than in the 100% wheat flour biscuits.

Conclusions

Based on the findings of this report, using OFSP and a haricot bean to wheat flour blend in biscuit formulation appears to be promising in terms of nutritional quality, acceptability, and cost. It is proposed that these products be marketed to vitamin A deficiency (VAD) customers as a newly established product to help mitigate food insecurity.

## 1. Introduction

Protein-energy malnutrition (PEM) is still a major health problem in developing countries and the leading cause of illness and death, especially among young children. ^1^ Malnutrition is estimated to be responsible for 60% of all deaths in children under the age of 5 years in developing countries, according to the World Health Organization. Nutritional progress is the most important factor in lowering high infant and under-5 years mortality rates, as well as ensuring children’s physical growth, social and mental development, and academic achievement. Protein-energy malnutrition is the leading cause of death in Sub-Saharan Africa, accounting for 25 to 35% of all deaths. ^1^ Ethiopia’s high malnutrition rates wreak havoc on the country’s economic and social development. ^2^ Protein-energy malnutrition, vitamin A deficiency, iodine deficiency disorders, and iron deficiency anemia are the most common types of malnutrition in Ethiopia. ^3^^,^^4^ Food-based policies are important for combating hunger and malnutrition, and attractive food characteristics include high nutrient density, low size, and the use of low-cost, locally available crops. Early adoption will be ensured at home and in the village. ^5^ Protein is essential for physical and mental growth in pre-school children and pregnant/lactating women. Animal-based foods are a decent source of protein, but they are costly. Pulses are a low-cost protein source, particularly for low-income families. Food-based approaches that are appropriate for the local settings are needed to combat PEM in rural areas.

People who live in rural areas have access to resources such as soil. They produce pulses and root and tuber crops on their farm but most of the time they sell the pulses. Since their children are not privileged to consume protein-source foods, they are susceptible to PEM. Pulses have several beneficial characteristics, including high nutritional value, long storage times, and a low cost compared with animal products. They contribute significantly to the provision of protein, electricity, and micronutrients to developing-world populations. ^6^

In terms of cultivation methods, uses, and the variety of conditions to which they have been adapted, pulses are a very diverse crop. Beans are high in dietary protein, which is important for human nutrition, particularly when combined with other foods. ^7^ Pulses are grown in various parts of Ethiopia; however, pulse consumption is not widespread in Ethiopia. The use of haricot bean flour in bakery products would open up a whole new market for protein-rich foods. In contrast to wheat alone, a good blend of wheat, OFSP, and haricot bean flours will produce nutritionally enriched biscuits.

## 2. Methods

### 2.1 Raw materials

OFSP of the Alamura variety was collected from CIP (International Potato Center). Soft wheat flour was obtained from the Hawassa flour factory, and haricot bean seed (Nasir) was obtained from the Hawassa Agricultural Research Center’s Southern Agricultural Research Institute.

### 2.2 Preparation of OFSP and haricot bean

The method described by Nshimiyimana ^8^ was used to prepare OFSP powder. OFSP were peeled by hand after being sorted and washed with tap water. Peeled OFSP were sliced with a slicer machine (Model-CL 30, robot @ couple) and blanched in a water bath at 65°C for 10 minutes. The treated slices were drained and dried for 24 hours in a 50°C oven. The dried OFSP flakes were ground into powder using a laboratory miller (Model R 23, robot @ couple) and sieved with a 500-m sieve scale. For further research, the powder was sealed in a polyethylene plastic bag and held in a cool, dark place for two months using a desiccator.

The method for preparing haricot bean flour was mentioned by Kaur and Kapoor. ^9^ The seed was sorted, washed, and soaked in distilled water in a water bath at 25°C for 24 hours at a ratio of 1:10 (w/v). The soaked seeds were washed twice in water, then rinsed with distilled water before being dried in a 60°C oven for 48 hours. The dried bean was dehulled and milled into flour using a laboratory grinder (Model R 23, robot @ couple) and sieved with a (500-m) sieve size before being packed in a polyethylene plastic bag for further study and held in a cool, dark place for two months using a desiccator.

### 2.3 Formulations of composite flour and recipes

The composite was created by taking into account some key facts about biscuit characteristics as well as previous research. Soft wheat flour is one of the most essential ingredients in biscuits, as flours that produce biscuits with a larger spread and smoother texture are preferred. ^10^ As a result, wheat flour was used as a major ingredient control in this analysis, with a lower limit of 40% in the composite flour formulation and the remaining 60% used for composite flour manipulation based on the previous studies. ^10^

### 2.4 Development of OFSP powder and haricot bean flour enriched biscuits

The instruments, baking process, and all other procedures needed for the experiment were all checked before the actual laboratory data collection. OFSP powder and haricot bean flour were blended into wheat flour at varying percentages of 5%, 10 %, 15%, 20%, 25%, and 30%. Biscuit dough was made following a commercial recipe and baking procedures. ^11^ Ingredients required to make biscuit were added in a similar amount to the different treatments in the experiment [baking powder (1.12 g)/100 g), cooking oil (28 g/100 g), sugar (5 g/100 g), salt (1 g/100 g), and 48 mL of water] were mixed thoroughly. The biscuit dough was made by hand, and the total time spent mixing was 20 minutes. After the dough was prepared, it was manually sheeted to a thickness of 5 mm sheeting. The biscuits were then formed and cut to a diameter of 48 mm before being placed on a lightly greased baking tray. In a baking oven, the biscuits were baked for 12 minutes at a temperature of 200°C. The baking temperature was chosen based on some key evidence, such as the fact that carotene is susceptible to heat degradation and that a temperature of 200°C for a 12-minute exposure period results in better beta-carotene retention.

### 2.5 Chemical analysis

***2.5.1 Functional properties of flours***

***Bulk density***

The bulk density of composite flour was determined using the method described by Oladele, and Aina. ^13^ In a 50-mL measuring cylinder, 10 g of sample flour was placed. The cylinder was tapped repeatedly until the volume remained unchanged. The sample’s bulk density (g/mL) was determined by dividing the weight of the sample by the volume of the sample.

$$\text{Bulk density}=\frac{\text{Mass of the sample}\;}{\text{Volume of sample}\;}$$ Eqn (1)

***Dispersibility***

The method described by Kulkarni *et al.* ^14^ was used to assess dispersibility. Water was applied to each volume of 100 mL after 10 g of flour sample was weighed into a 100-mL measuring cylinder. The setup was vigorously stirred and left for three hours. The number of settled particles was measured and deducted from a total of 100. The percentage dispersibility of the differences was recorded.

% of dispersibility = 100−the volume of settled particle Eqn (2)

***Water absorption capacity (WAC)***

According to Aremu *et al.*, ^15^ the water absorption ability of flour samples was determined. In a centrifuge tube, 1 g of flour sample was mixed with 10 mL of distilled water and allowed to stand at room temperature for 30 minutes. The supernatant was collected in a 10 mL graduated cylinder after centrifugation at 5,000 rpm for 30 minutes. The amount of oil absorbed per gram of flour sample was measured as mL of oil absorbed per gram of flour sample.

***Oil absorption capacity (OAC)***

According to Aremu *et al.*, ^15^ the oil absorption ability of flour samples was determined. In a centrifuge tube, 1 g of flour sample was mixed with 10 mL of oil and allowed to sit at room temperature for 30 minutes. It was centrifuged for 30 minutes at 5,000 rpm, with the supernatant collected in a 10 mL graduated cylinder. The amount of oil absorbed per gram of flour sample was measured as mL of oil absorbed per gram of flour sample.

***2.5.2 Proximate analyses for flour and biscuit***

The standard methods of the Association of Official Analytical Chemists ^16^ were used to assess proximate analyses. The Kjeldahl approach was used to calculate total nitrogen (TN). The crude protein content was measured by multiplying TN by a conversion factor of 6.25 (% protein=TN×6.25), and the crude fat, crude fiber, ash content, and moisture content of the sample were determined according to the Association of Official Analytical Chemists. ^16^ The difference between 100 and (ash+protein+fiber+fat+moisture) was used to calculate the utilizable carbohydrate material. Using Atwater’s conversion factors, the energy content in kcal/100 g was calculated by multiplying the percentages of crude fat, crude protein, and carbohydrate by factors of 9, 4, and 4, respectively.

***2.5.3 Determination of physical properties of biscuit***

To determine the spread factor (SF), the diameter (D) and thickness (T) of the biscuit were calculated using different methods. ^17^ The diameters of the biscuits were determined by putting six cookies horizontally edge-to-edge and rotating at a 90° angle for a duplicate measurement. The thickness of biscuits was determined by piling six cookies on top of one another, then taking a duplicate reading by shuffling the biscuits. All of the measurements were performed in two replicates of six cookies each, and the values per biscuit were calculated by dividing the total readings by six.

SF = D/T Eqn (3)

***2.5.4 Sensory acceptability***

Panelists measured the sensory acceptability of biscuits based on their willingness to engage in the study. ^18^ 30 consumer panelists were chosen at random from university food science and nutrition students to test sensory characteristics of the products, such as appearance, taste, scent, crispiness, color, flavor, and overall acceptability. The researchers used five-point hedonic scales (1 = very much hate, 2 = dislike, 3 = neither like nor dislike, 4 = like, and 5 = like very much).

***2.5.5 Statistical data analysis***

For nutritional analysis, a randomized design (CRD) was used, and for sensory analysis, a randomized controlled block design (RCBD) was used. The knowledge was examined twice. SAS analytical software version 9.0 ( RRID:SCR_008567) was used to analyze the data using one-way analysis of variance (ANOVA), JASP ( RRID:SCR_015823) is an open access alternative that can be used to perform analysis. A p-value <0.05 was used to quantify substantial differences in the list. The data were presented as a mean with a standard deviation.

## 3. Results and discussion

### 3.1 Functional properties of OFSP powder, wheat, and haricot bean flours

The bulk density, water absorption ability, oil absorption capacity, and dispersibility of OFSP powder, wheat, and haricot bean flours are shown in Table 1. OFSP powder had a bulk density that was slightly higher than wheat and haricot bean flours. However, the water absorption capacity, oil absorption capacity, and dispersibility of the three flours were found to be substantially different from each other. Wheat flour had the highest while OFSP powder had the lowest oil absorption capacity and dispersibility. Wheat flour had the lowest water absorption potential, while haricot bean flour had the highest.

**Table 1.** Functional properties OFSP powder, wheat, and haricot bean flours.

Flour sample	BD (g/mL)	WAC (%)	OAC (%)	DESP (%)
WF	0.74 ± 0.03 ^b^	16.2 ± 0.56 ^c^	21.3 ± 0.14 ^a^	77.5 ± 2.12 ^a^
HBF	0.86 ± 0.01 ^b^	23.9 ± 0.98 ^a^	18.7 ± 0.14 ^b^	65.5 ± 0.70 ^b^
OFSPF	1.05 ± 0.06 ^a^	19.2 ± 0.70 ^b^	17.85 ± 0.35 ^c^	45.5 ± 2.12 ^c^

Where, BD = Bulk density, WAC = Water absorption capacity, OAC = Oil absorption capacity, DESP = Dispersibility, WF = Wheat flour, HBF = Haricot bean flour, OFSPF = Orange fleshed sweet potato flour. Values with the same column with different superscript letters are significantly different from each other (p<0.05) and values are averages of duplicate readings (mean ± SD, n = 2).

In this analysis, the bulk density of OFSP (Alamura variety) powder is higher than that recorded by Tiruneh ^19^ for the Kulfo and Tulla varieties (0.74–0.62). The bulk density of haricot bean flour is higher than that of red kidney bean flour (0.51–0.55 g/mL), as stated by Khalil *et al*. ^20^ Wheat flour has a bulk density of 0.75 g/mL, which is almost identical to that stated by Biniyam. ^21^ This disparity may be due to varietal differences. The bulk density of flours is calculated by particle size and initial moisture content, according to the current research. ^22^ The high bulk density of flour suggests that it is suitable for use in food preparations; however, the low bulk density of complementary foods would be advantageous. ^23^ Both haricot bean flour and OFSP powder have a higher bulk density than wheat flour, so adding haricot bean and OFSP to the composite flour would increase the bulk density of the composite flour as compared to wheat flour. The manufacture of confectioneries such as cakes, sweet pastries, doughnuts, and cookies benefits from an increase in flour bulk density. ^24^ This means that the flour’s heaviness and suitability for confectionery production are both positive. A rise in bulk density improves packaging performance. As a result, a larger amount may be packed into a smaller volume. ^25^

The water absorption ability of haricot bean flour was higher than the value (17.3–16.8%) recorded by Shimelis *et al.* ^26^ on various haricot bean flour varieties. The WAC of the OFSP powder is lower than the values recorded by Tiruneh ^19^ for the Kulfo and Tulla varieties. The WAC of wheat flour is much higher than the value recorded by Biniyam ^21^ (8.5%). Many hydrophilic elements, such as carbohydrates and proteins (polar amino acid residues), have a strong affinity for water and contribute to the high WAC value. ^27^ Water absorption is reduced in flours with a lower proportion of polar amino acids and a higher proportion of nonpolar amino acids. ^28^ As a result, the observed differences in different flours may be due to differences in protein concentration, water interaction, and conformational characteristics. ^29^

Both haricot bean flour and OFSP powder have a higher WAC than wheat flour, so adding haricot bean and OFSP to the composite flour would improve the WAC of the composite flour compared with wheat flour. Increased water absorption weakens the dough and causes it to lose its development and stability. The ability to absorb water is essential in product consistency and bulking, as well as in baking applications. ^30^ Water absorption capacities are closely associated with cookie spread, according to Doescher *et al.*, ^31^ as cited by Vieira *et al.*; ^32^ higher water absorption capacities may have contributed to the lower spread ratio. The oil absorption of wheat flour is higher than the values recorded by Baljeet *et al.* ^33^ and Suresh and Samsher ^24^ (16.9% and 14.6%, respectively). By a factor of, ^26^ the oil absorption ability of haricot bean flour (18.7%) is lower than the values observed for various haricot bean varieties (24.9–35.2%). The oil absorption ability of OFSP powder in this study is comparable to the 16.8–18.4% recorded by Tiruneh ^19^ for Tulla and Kulfo varieties.

Wheat flour has a higher oil absorption ability than OFSP powder and haricot bean flour, owing to the lipophilic quality of its constituents. ^34^ Protein conformation, amino acid composition, and surface polarity or hydrophobicity all play a role in lipophilicity. ^24^ Wheat flour has a higher OAC than OFSP powder and haricot bean flour, so adding haricot bean and OFSP to the composite flour would lower the OAC compared to wheat flour. When the OAC of composite flour is reduced, the taste and mouthfeel of the cookies suffer. Because of the flour’s higher oil absorption potential, it’s perfect for enhancing flavor and mouthfeel when used in food preparation. ^24^ Dispersibility is a key metric for determining how well flour or flour blends will rehydrate with water without forming lumps. Wheat flour had a substantially higher dispersibility than haricot bean and OFSP powder in this report. The higher the dispersibility, the stronger the reconstitution property and it’s what’s used to make fine dough consistency during mixing. The property of dispersibility also defines flour’s propensity to detach from water molecules, exposing its hydrophobic behavior. Wheat flour has a higher dispersibility than OFSP powder and haricot bean flours, and the addition of haricot bean and OFSP will reduce the composite flour’s dispersibility as compared to wheat flour. Significantly lower dispersibility of composite flour contributes to decreased dough consistency during mixing as a result of this finding.

### 3.2 Proximate composition of OFSP powder, wheat, and haricot bean flours

Table 2 shows the proximate composition of OFSP powder, wheat, and haricot bean flours (moisture, ash, crude protein, crude fat, crude fiber, carbohydrate, and energy). Moisture, protein, ash, fiber, and carbohydrate content were found to be substantially different between the three flours. Haricot bean flour has a slightly higher fat content than wheat flour and OFSP powder, which did not vary significantly. Wheat flour had the highest moisture content, while OFSP powder had the lowest. Haricot bean flour had the most protein, ash, fiber, and fat, while OFSP powder had the least protein and wheat flour had the least ash, fiber, and fat. The highest carbohydrate content was contained in OFSP powder, followed by wheat flour. Furthermore, the energy content of OFSP powder was substantially higher than that of haricot bean flour.

**Table 2.** Proximate composition of OFSP powder, wheat, and haricot bean flours (as is basis).

Flour	Moisture (%)	Crude Protein (%)	Crude Ash (%)	Crude Fiber (%)	Crude Fat (%)	CHO (%)	Energy (kcal/g)
WF	10.16 ± 0.07 ^a^	11.02 ± 0.60 ^b^	1.16 ± 0.08 ^c^	1.16 ± 0.07 ^c^	1.66 ± 0.32 ^b^	74.66 ± 1.07 ^b^	357.70 ± 1.64 ^ab^
HBF	8.49 ± 0.23 ^b^	18.43 ± 1.38 ^a^	3.83 ± 0.70 ^a^	4.99 ± 0.20 ^a^	2.66 ± 0.14 ^a^	61.58 ± 2.38 ^c^	344.04 ± 7.27 ^b^
OFSPF	4.49 ± 0.23 ^c^	5.25 ± 0.56 ^c^	2.16 ± 0.08 ^b^	4.16 ± 0.07 ^b^	1.33 ± 0.00 ^b^	82.51 ± 0.92 ^a^	363.04 ± 1.42 ^a^

Where, WF = Wheat flour, HBF = Haricot bean flour, OFSPF = Orange fleshed sweet potato flour. Values with the same column with different superscript letters are significantly different from each other (p < 0.05) and values are averages of duplicate readings (mean ± SD, n = 2).

In terms of protein, ash (mineral), fiber, and fat content, haricot bean flour appears to have an advantage over wheat flour. The addition of OFSP powder appears to improve the fiber and ash content as well. According to Biniyam, ^21^ the product contains 13% moisture, 11% protein, 0.69% ash, 2% crude fiber, 2% fat, 71.3% carbohydrate, and 347.2 kcal/g energy. This research had higher moisture, crude fiber, and fat content than the current study, comparable protein content, but lower carbohydrate and energy content than the current study. Varietal disparities and measurement processes may be to reason for these variations.

For eight different haricot bean varieties grown in Ethiopia, recorded 11.1–11.4%moisture, 18.0–22.1% protein, 2.9–4.3% ash, 4–5.9% crude fiber, 1.3–2.8% fat, 56.5–61.6% carbohydrate, and 330–343.2 kcal/g energy content. ^35^ Except for the higher moisture content of this sample, which may be attributed to drying methods or determination methods, all of the findings are consistent with the current study. For OFSP powder pretreated and dried with various methods, recorded 4–8% moisture, 4–5.8% protein, 4.2–7.5% ash, 3.7–7.3% crude fiber, 0.9–2% fat, and 80–83.7% carbohydrate content. ^36^ Except for the lower ash content in this study, these findings are consistent with the current study. This inconsistency may be due to discrepancies in varietals, drying methods, and measurement methods.

### 3.3 Effect of blending ratio of composite flour on proximate composition of biscuits

Table 3 shows the approximate compositions of biscuits (moisture, ash, crude protein, crude fat, crude fiber, carbohydrate, and energy). The moisture, protein, ash, fiber, and fat content of the biscuit increased as the percentage of haricot bean flour and OFSP powder increased, as shown in Table 3. However, when 15% haricot bean flour and 15% OFSP powder are added to 100% wheat flour biscuits, the rise in proximate composition becomes most noticeable (control). However, when compared with the other proximate elements, carbohydrate content, and energy value showed the opposite trend. As the proportion of wheat flour was reduced and the proportion of haricot bean and OFSP powder was increased, both carbohydrate content and energy value decreased. The addition of 15% haricot bean flour and 15% OFSP powder resulted in a significant decrease in carbohydrate content, whereas the addition of 25% haricot bean flour and 25% OFSP powder resulted in a significant decrease (p < 0.05) in energy value.

**Table 3.** Effect of blending ratios on biscuits proximate composition on a wet weight basis (%).

Blends	Moisture (%)	Crude Protein (%)	Crude Ash (%)	Crude Fiber (%)	Crude Fat (%)	CHO (%)	Energy (kcal/g)
W100%	6.99 ± 0.02 ^e^	11.69 ± 0.14 ^e^	1.99 ± 0.02 ^d^	1.49 ± 0.12 ^e^	1.71 ± 0.06 ^e^	76.14 ± 0.41 ^a^	366.53 ± 1.34 ^a^
W90%, H5%, OF5%	7.16 ± 0.07 ^e^	12.3 ± 0.43 ^e^	2.16 ± 0.11 ^cd^	1.83 ± 0.12 ^de^	1.74 ± 0.07 ^e^	74.8 ± 0.19 ^a^	364.10 ± 4.13 ^a^
W80%, H10%, OF10%	7.49 ± 0.04 ^de^	13.21 ± 0.27 ^de^	2.33 ± 0.02 ^c^	2.16 ± 0.45 ^cd^	1.87 ± 0.15 ^de^	72.93 ± 0.93 ^ab^	361.41 ± 5.55 ^ab^
W70%, H15%, OF15%	8.0 ± 0.15 ^cd^	14.02 ± 0.25 ^cd^	2.66 ± 0.09 ^b^	2.5 ± 0.21 ^c^	1.99 ± 0.04 ^cd^	70.82 ± 2.58 ^bc^	357.33 ± 6.18 ^ab^
W60%, H20%, OF20%	8.5 ± 0.42 ^c^	15.22 ± 1.08 ^bc^	2.83 ± 0.03 ^b^	3.16 ± 0.05 ^b^	2.16 ± 0.11 ^bc^	68.12 ± 1.97 ^cd^	352.82 ± 0.48 ^ab^
W50%, H25%, OF25%	9.33 ± 0.45 ^b^	16.07 ± 0.90 ^ab^	3.16 ± 0.19 ^a^	3.66 ± 0.02 ^b^	2.33 ± 0.02 ^ab^	65.45 ± 0.72 ^d^	347.05 ± 12.36 ^bc^
W40%, H30%, OF30%	10.96 ± 0.09 ^a^	17.15 ± 1.01 ^a^	3.33 ± 0.10 ^a^	4.33 ± 0.24 ^a^	2.49 ± 0.01 ^a^	61.73 ± 1.69 ^e^	337.93 ± 5.81 ^c^

Where, W = Wheat flour, H = Haricot bean flour, OF = Orange fleshed sweet potato flour. Values with the same column with different superscript letters are significantly different from each other (p < 0.05) and values are averages of duplicate readings (mean ± SD, n = 2).

Since OFSP powder and haricot bean flour have a higher water absorption ability and fiber content than wheat flour, the increase in moisture content in this study may be due to increasing the percentage of OFSP powder and haricot bean flour to wheat flour. This study confirms the results of Biniyam, ^21^ which found that cookies made with wheat, quality protein maize, and carrot composite flour retained more moisture than wheat flour. The latter two flours have higher fiber and water absorption ability than wheat flour.

According to Khaliduzzaman *et al.*, ^37^ they replaced wheat flour with 20% potato flour while making biscuits, and the addition of potato flour increased the moisture content. The results of this study agree with those of Vieira, ^32^ who found that blending wheat flour with residue from king palm processing, which has a higher fiber content than wheat flour, resulted in higher fiber content than wheat flour. The hydroxyl group present in the fiber structure allows higher total fiber in non-wheat flour to interact reasonably well with a large amount of water, according to Wang *et al.* ^38^ Since haricot bean flour has higher protein content than wheat flour, the rise in protein content in this study may be due to switching from haricot bean flour to wheat flour. The current study’s findings are consistent with Abayomi *et al.* ^39^ who recorded increased protein content in cookies made with sweet potato and fermented soybean flours, with the percentage of fermented soybean flour increasing the protein content of cookies. Cookies made with 30% soybean supplementation had a high protein content of 21.65%, while cookies made with 100% sweet potato flour had lower protein content.

According to Ndife *et al*., ^40^ increased protein content in cookies from wheat and soya bean flours where increasing the percentage of soya bean flour increased the protein content of cookies ranged 8.75 to 24.65%. Similar findings were reported in a research study that showed an improvement in protein content in biscuit production from cassava-wheat-bambara flour blends with corresponding increases in the proportion of bambara flour supplementation. ^41^ Since haricot bean flour and OFSP powder have higher ash content than wheat flour, the increase in ash content of biscuits in this study may be due to increasing the percentage of haricot bean flour and high dry matter content of OFSP powder to wheat flour.

This result in cookies made with sweet potato and fermented soybean flours showed that raising the percentage of soya bean flour raised the ash content in cookies, with cookies made with 30% soybean supplementation having a high ash content of 2.57% and cookies made with 100% sweet potato powder having a lower ash content of 2.20%. ^39^ The current study’s findings are consistent with those of Ndife *et al.*, ^40^ who found that raising the percentage of soya bean flour in cookies increased the ash content of cookies by 2.15–2.95%, which is lower than the current study’s findings. Similar findings were reported increasing the amount of ash content in cookies made from wheat and OFSP powder composite flours by increasing the percentage of OFSP powder in the composite flour by 1.05–1.17%, which is lower than the current research. ^42^ Since OFSP powder and haricot bean flour have higher fiber content than wheat flour, the increase in fiber content in this study may be due to increasing the percentage of OFSP powder and haricot bean flour to wheat flour.

The result of the current study is consistent with Ndife *et al*. ^40^ who found that increased fiber content in cookies from wheat and soya bean flours where increasing the percentage of soya bean flour increase the fiber content of cookies ranged by 3.29–5.73%. Biniyam ^21^ recorded higher fiber content in cookies made with wheat, quality protein maize, and carrot composite flours, the latter two having more fiber than wheat flour. The results of this study agree with those of Vieira *et al.*, ^32^ who found that blending wheat flour with residue from king palm processing, which has a higher fiber content than wheat flour, resulted in higher fiber content than wheat flour. Increased fiber content can help with waste passage by expanding the inside walls of the colon, making anti-constipation more efficient, lowering cholesterol levels in the blood, and lowering the risk of various cancers. ^43^

Since haricot bean flour has a higher fat content than wheat flour, the rise in crude fat content in this study may be attributed to increasing the ratio of haricot bean flour to wheat flour. The current results are consistent with Abayomi *et al.* ^39^ that showed an improvement in fat content in cookies made with sweet potato and fermented soybean flours while the amount of soya bean flour was increased. Cookies made with 30% soybean supplementation had a high fat content of 5.25%, while cookies made with 100% sweet potato flour had a lower fat content of 1.22%. This finding was made in a research study by Biniyam ^21^ who found that cookies made with wheat, quality protein maize, and carrot composite flours had higher fat content than wheat flour. The current study’s findings are consistent with Ndife *et al.* ^40^ reports of increased fat in cookies made with wheat and soya bean flours, where increasing the percentage of soya bean flour increased the fat content of cookies by 4.50–7.13%. Furthermore, Singh *et al.* ^44^ recorded a similar pattern in biscuit production from cassava-wheat-Bambara flour blends, with an increase in fat content and a corresponding increase in the proportion of Bambara flour supplementation.

The decrease in carbohydrate content of the cookie may be due to a rise in moisture, fat, ash, and fiber content as the proportion of OFSP powder and haricot bean flour in the formulation was increased, resulting in a decrease in carbohydrate content because carbohydrate is measured by difference. The current result was consistent with the results of Biniyam, ^21^ who found that cookies made from wheat, quality protein maize, and carrot composite flour had lower carbohydrate content and was higher in moisture, fat, ash, and fiber content. Furthermore, Vieira *et al.* ^32^ reported a reduction in carbohydrate content of cookies by combining wheat flour with residue from king palm processing, which has a higher fiber, ash, and fat content than wheat flour. The energy content of the cookies follows the pattern of the carbohydrate content, as carbohydrate is the primary source of energy throughout the cookies. Based on Singh *et al.*, ^44^ the recommended minimum daily energy requirement for an average Ethiopian man is 1820 kilocalories per person per day. Biscuits made in this study can provide about 18.56–20.13% of an average man’s daily energy requirements, with higher protein, mineral, and fiber content than wheat-based biscuits.

### 3.4 Effect of blending ratio of composite flour on physical characteristics of biscuits

Table 4 displays the physical characteristics of biscuits (diameter, thickness, and spread factor). The physical characteristics of biscuits did not improve significantly (p < 0.05) when haricot bean flour and OFSP powder were added. However, when compared with the control, the diameter, thickness, and spread factor decrease slightly when the proportion of haricot bean flour and OFSP powder is increased. The only exception was the diameter of biscuits produced with 40% wheat, 30% haricot bean flour, and 30% OFSP powder, which was slightly lower (p < 0.05) than the control (100% wheat flour).

**Table 4.** Effect of blending ratios on physical characteristics of biscuits.

Blends	Diameter (cm)	Thickness (cm)	Spread factor
W100%	4.87 ± 0.07 ^a^	0.51 ± 0.77 ^a^	9.56 ± 1.29 ^a^
W90%, H5%, OF5%	4.77 ± 0.03 ^ab^	0.50 ± 0.77 ^ab^	9.55 ± 1.38 ^a^
W80%, H10%, OF10%	4.65 ± 0.07 ^ab^	0.49 ± 0.77 ^a^	9.50 ± 1.33 ^a^
W70%, H15%, OF15%	4.54 ± 0.21 ^ab^	0.48 ± 0.77 ^a^	9.45 ± 1.06 ^a^
W60%, H20%, OF20%	4.44 ± 0.36 ^ab^	0.47 ± 0.77 ^a^	9.41 ± 0.78 ^a^
W50%, H25%, OF25%	4.32 ± 0.45 ^ab^	0.46 ± 0.77 ^a^	9.34 ± 0.57 ^a^
W40%, H30%, OF30%	4.14 ± 0.34 ^b^	0.45 ± 0.70 ^a^	9.26 ± 0.68 ^a^

Where, W = wheat flour, H = Haricot bean flour, OF = orange fleshed sweet potato flour.

Values with the same column with different superscript letters are significantly different from each other (p < 0.05) and values are averages of duplicate readings (mean ± SD, n = 2).

With the addition of OFSP, different studies recorded a decrease in the diameter, ^44^^,^^42^ thickness, ^44^^-^^46^ and spread factor ^42^^,^^47^ of wheat cookies. These findings are consistent with the fact that the addition of haricot bean flour and OFSP powder reduced the diameter, thickness, and spread ratio of biscuits in the current report. The addition of haricot bean flour in addition to the OFSP powder may have resulted in a small decrease in the diameter, thickness, and spread ratio of the biscuits in this report. The haricot bean’s higher protein content may have some gluten-substituting properties. The haricot bean’s higher dispersibility than OFSP may have a secondary effect of lowering the spread ratio. Rapid partitioning of free water to hydrophilic sites during mixing increased dough viscosity, restricting biscuit spread. ^48^ As a result of the addition of OFSP powder and haricot bean flour to cookies, the distribution of the cookies is reduced.

### 3.5 Effect of blending ratio of composite flour on sensory acceptability of biscuits

Table 5 demonstrates the sensory acceptability of biscuits (color, shape, flavor, taste, texture, crispiness, and overall acceptability). Except for the one with the highest haricot bean flour and OFSP powder (W40%, H30%, OF30%), the appearance of the composite flour biscuits did not display a substantial difference (p < 0.05) compared with 100% wheat flour biscuits (control). The color of all the composite biscuits did not vary significantly from the control (p < 0.05). The crispiness of composite flour biscuits containing 20, 25, and 30% haricot bean flour and OFSP powder was slightly lower (p < 0.05) than that of biscuits made entirely of wheat. All of the composite flour biscuits, except W60%, H20%, OF20%, and W40%, H30%, OF30%, tasted and were accepted in the same way as the 100% wheat flour cookies in terms of flavor, taste, and overall acceptability. In comparison with 100% wheat flour biscuits, the appearance, color, flavor, taste, and overall acceptability scores of the biscuits improved with the addition of haricot bean flour and OFSP powder up to H20%, OF20%. The crispiness of the biscuits, on the other hand, decreased as haricot bean flour and OFSP powder was added. Despite this, the crispiness of the composite flour biscuits is not substantially different from the control up to the addition of 15% haricot and 15% OFSP.

**Table 5.** Effect of blending ratios on sensory properties of biscuits.

Blends	Appearance	Color	Flavor	Crispiness	Taste	OAA
W100%	4.11 ± 0.80 ^ab^	4.08 ± 0.84 ^a^	3.73 ± 1.05 ^b^	4.26 ± 1.05 ^a^	3.85 ± 1.11 ^b^	3.98 ± 0.65 ^b^
W90%, H 5%, OF5%	4.21 ± 0.76 ^ab^	4.10 ± 0.75 ^a^	3.88 ± 0.84 ^ab^	4.25 ± 0.85 ^a^	3.91 ± 0.92 ^b^	4.00 ± 0.68 ^ab^
W80%, H10%, OF10%	4.28 ± 0.55 ^a^	4.16 ± 0.78 ^a^	3.90 ± 0.75 ^ab^	4.16 ± 1.06 ^a^	3.95 ± 0.81 ^ab^	4.05 ± 0.74 ^ab^
W70%, H15%, OF15%	4.30 ± 0.80 ^a^	4.18 ± 0.74 ^a^	3.98 ± 0.91 ^ab^	4.06 ± 1.00 ^ab^	3.98 ± 0.96 ^ab^	4.13 ± 0.59 ^ab^
W60%, H20%, OF20%	4.36 ± 0.75 ^a^	4.23 ± 0.78 ^a^	4.10 ± 0.93 ^a^	3.70 ± 1.09 ^bc^	4.25 ± 0.77 ^a^	4.25 ± 0.75 ^a^
W50%, H25%, OF25%	3.93 ± 0.91 ^a^	3.96 ± 0.95 ^a^	3.71 ± 0.86 ^b^	3.60 ± 1.09 ^c^	3.75 ± 1.05 ^bc^	3.91 ± 0.92 ^b^
W40%, H30%, OF30%	3.41 ± 1.10 ^c^	3.51 ± 1.12 ^a^	3.21 ± 0.94 ^c^	3.51 ± 1.09 ^c^	3.43 ± 0.76 ^c^	3.50 ± 0.79 ^c^

Where, W = Wheat flour, H = Haricot bean flour, OF = Orange fleshed sweet potato flour, OAA = Overall acceptability Values with the same column with different superscript letters are significantly different from each other (p < 0.05) and values are averages of duplicate readings (mean ± SD, n = 2).

The most critical quality attributes that can affect the acceptability of a food product and a consumer’s buying decision are its appearance and color. ^49^ The current study’s appearance results are consistent with the findings of, ^50^ which found that biscuits made from composite flours containing 70% wheat flour and 30% OFSP powder had a higher appearance value. The attractive color of OFSP ^51^ may have contributed to the composite flour biscuits’ increased color acceptability. The color acceptability of the current study agrees with the results of, ^50^ who found that biscuits made with 70% wheat and 30% OFSP composite flours had higher color acceptability. Similarly, ^51^ discovered that the color acceptability pattern of biscuits made from composite wheat flour and OFSP powder increased over time. The addition of haricot bean flour and OFSP powder increased the taste acceptability score of the cookies, which may be attributed to the sweetness of the OFSP powder.

The current study’s taste results are consistent with the findings of Afework *et al.*, ^50^ who recorded a higher taste acceptability score for biscuits made with 70% wheat and 30% OFSP composite flours. The taste acceptability score of biscuits made from wheat and OFSP composite flours improved in the same way, according to Onabanjo and Ighere. ^51^ A food product’s flavor is a mixture of taste and aroma, as well as other sensory qualities. The improvement in the flavor of the cookies with increased haricot bean and OFSP may be attributed to the sweetness of the OFSP, similar to the taste attribute. The same trend of increasing flavor acceptability score was observed in flatbread made from wheat and OFSP composite flours, according to Terefe. ^52^ The crispiness is one of the most important textural characteristics of dry snack foods, indicating freshness and high quality. A crisp product should, in general, be strong and snap easily when bent, emitting a crunchy sound. ^53^ The decrease in biscuit crispiness as the proportion of haricot bean flour and OFSP powder is increased may be attributed to the increased moisture content of the biscuits as the proportion of haricot bean flour and OFSP powder is increased.

The decrease in biscuit crispiness as the proportion of haricot bean flour and OFSP powder is increased may be attributed to the increased moisture content of the biscuits as the proportion of haricot bean flour and OFSP powder is increased. According to Manley, ^54^ a biscuit’s structure will not be crisp at higher moisture levels. Finally, overall acceptability is a metric that assesses a product’s overall acceptance. Except for crispiness, the overall acceptability of the biscuits improved as the haricot bean flour and OFSP powder content increased. With the addition of OFSP powder to wheat flour, Onabanjo and Ighere ^51^ recorded an improvement in overall biscuit acceptability. In the end, all of the composite flour biscuits, except for the W40%, H30%, and OF30% were as good as or better than the 100% wheat biscuits in terms of acceptability.

## Conclusion

Biscuits with greater sensory acceptability than wheat-based biscuits can be made by substituting haricot bean flour and OFSP powder for up to 40% of the wheat in the recipe (20% haricot bean and 20% OFSP). Biscuits made with haricot bean flour and OFSP powders are more nutritious than wheat-based cookies in terms of proximate composition (high in protein, fiber, and ash (mineral). Physical parameters such as diameter thickness and spread factor measurements of biscuits with more OFSP supplementation were found to be less bulky, reducing packaging, transportation, and distribution costs. Based on the findings of this report, using OFSP and a haricot bean to wheat flour blend in biscuit formulations appears to be promising in terms of nutritional quality, acceptability, and cost. Development of nutritious and more sensory appealing biscuits by replacing wheat with locally available and inexpensive ingredients (haricot bean and OFSP) will benefit high-yielding native plant species, provide a better supply of the nutrient-rich commodity, and improve the overall use of domestic agriculture production.

## Data availability

OSF Register: Underlying data for ‘Effect Of Blending Ratio Of Wheat, Orange Fleshed Sweet Potato Powder and Haricot Bean Flour on Proximate Composition, Physical Properties and Sensory Acceptability of Biscuits’; https://doi.org/10.17605/OSF.IO/4SP9A.

Data are available under the terms of the Creative Commons Attribution 4.0 International license (CC-BY 4.0).
