# Effect of blending ratio of wheat, orange fleshed sweet potato and haricot bean flour on proximate compositions, β-carotene, physicochemical properties and sensory acceptability of biscuits’

**DOI:** 10.12688/f1000research.52634.2

**Published:** 2022-03-11

**Authors:** Fieben Kindeya, Welday Hailu, Tilku Dessalegn, Gesessew L. Kibr

**Affiliations:** 1 Department of Food and Nutritional Sciences, Shambu Campus, Wollega University, Shambu Town, Oromia Region, 38, Ethiopia; 2 School of Nutrition, Food Science and Technology, Hawassa University, Hawassa, Sidama Region, Ethiopia

**Keywords:** Protein-Energy Malnutrition; Biscuit; Haricot bean; Sensory acceptability

## Abstract

**Background: **Protein-energy malnutrition and vitamin A deficiency (VAD) are the most important public health issues, and a food-based strategy is crucial to combat those health problems among the vulnerable group of people.

**Methods: **Composite biscuits were made with 100:0:0, 90:5:5, 80:10:10, 70:15:15, 60:20:20, and 50:25:25 percent wheat, haricot bean, and orange-fleshed sweet potato (OFSP) flours.Standard methods were used to evaluate the proximate compositions, β-carotene, physical properties, functional properties, and sensory acceptability. A one-way analysis of variance model was used to statistically evaluate the data using the statistical analysis system software package, version 9.0 standard methods.

**Results: **The results showed that partially replacing wheat with haricot beans and OFSP increased the β-carotene and proximate compositions significantly. When wheat was replaced with haricot beans and OFSP, the physical characteristics of the biscuits did not vary significantly from those of biscuits made entirely of wheat flour. Sensory acceptability (appearance, color, flavor, taste and overall acceptability) was higher in the composite biscuits with up to 40% wheat substitution than in the 100% wheat flour biscuits.

**Conclusion: **Based on the findings of this report, replacing wheat with OFSP and haricot beans in biscuit formulation appears to be promising in improving nutritional quality, sensory acceptability, and beta carotene. It is proposed that these products can mitigate food insecurity and deficiency of vitamin A.

## 1. Introduction

Protein malnutrition and vitamin deficiencies are the most prevalent and serious public health issues, particularly among pregnant and lactating mothers, as well as children. ^1^ ^,^ ^2^ VAD is a major public health issue in Ethiopia. ^2^ In countries where people eat a monotonous diet, VAD has always been a severe problem. ^3^ Food-based policies are important for combating malnutrition, and attractive food characteristics include high nutrient density, low size, and the use of low-cost, locally available crops. Early adoption will be ensured at home and in the village and is essential for physical and mental growth. ^4^

In Sub-Saharan Africa, especially among resource-poor households, diets are often low in variety and dominated by staple crops such as maize, rice, cassava, sorghum, millet, bananas, and sweet potatoes, all of which are low in micronutrients, resulting in widespread micronutrient deficiencies. ^5^ Root crops that contain pro-vitamins have been shown to be an effective way to treat and reduce VAD. OFSP ( *Ipomoea Batatas L.*) is a naturally bio-fortified crop and it has great potential to be used in food-based intervention programs, especially in developing countries, to address VAD, which causes illness and death and is considered a significant dietary resource of VA carotenoids and non-provitamin A carotenoids. ^6^ ^–^ ^9^ The introduction of OFSP in an integrated agriculture and nutrition intervention resulted in improvements in VA stored in the liver, dietary VA intake, and serum retinol concentrations. ^10^ ^,^ ^11^ An impact case study suggested that replacing the current white-fleshed varieties with new orange-fleshed varieties that are high in β-carotene content would benefit individuals who are currently at risk of VAD. ^12^ However, animal and plant VA-rich foods are only seasonally available, unpalatable to children, and often absent from the diets of low-income households. OFSP is an exception crop that is a promising solution to VAD because it is high in beta-carotene and absorbs it much better than other leaves and vegetables. ^13^ It can give energy and vitamins and minerals for our daily needs, such as β-carotene, thiamin, iron, and vitamin C. However, it is often low in protein, ranging from 1-8.5%. ^14^ ^,^ ^15^

Compared to animal-based foods, pulses, in general, an important food category for humans, have high protein content, and thus, can help to improve the protein content of meals, especially for low-income households. ^16^ The protein content of most pulses is between 20-30 g/100 g, twice as much protein as cereals. ^17^ They are also rich sources of complex carbohydrates, vitamins, and minerals. ^18^ Biscuits made from wheat flour are one of the most widely-eaten consumer products in the world. They are an affordable product, and they have good taste and a long shelf life. ^19^ Unfortunately, biscuits usually contain high levels of easily digested starch, sugar, and butter, and low levels of dietary fiber, which nutritionists suggest makes them a rather unhealthy constituent of our diet. Bakers are well aware of these issues, and they have shown some interest in developing biscuits, which can be regarded as functional foods containing less butter. ^20^ ^–^ ^22^ So far, bakery research has attempted to make more healthy products by incorporating new ingredients into biscuit mixes to increase their nutritional and textural qualities. ^19^ In considering the development of a new product, it is important to use locally sourced ingredients whose tastes are appreciated by the ethnic groups the products are intended for. ^23^ This study aimed to investigate formulations for a series of biscuits using novel ingredients, including OFSP and haricot bean ( *Phaseolus vulgaris L.*) flour. The aim was to produce a balanced, low-cost, and healthy snack product. Considering the potential health benefits of OFSP and haricot bean flour and the increasing consumption of healthy foods, the objective of the present study was to prepare nutritious biscuits to deliver a nutritious and healthy product.

## 2. Methods

### 2.1 Raw materials

An orange-fleshed sweet potato of the Alamura variety was collected from Hawassa International Potato Center, Ethiopia. Soft wheat flour was obtained from the Hawassa flour factory, and haricot bean seed (Nasir) was obtained from the Hawassa Agricultural Research Center’s Southern Agricultural Research Institute.

### 2.2 Preparation of OFSP and haricot bean flour

**Figure 1.**
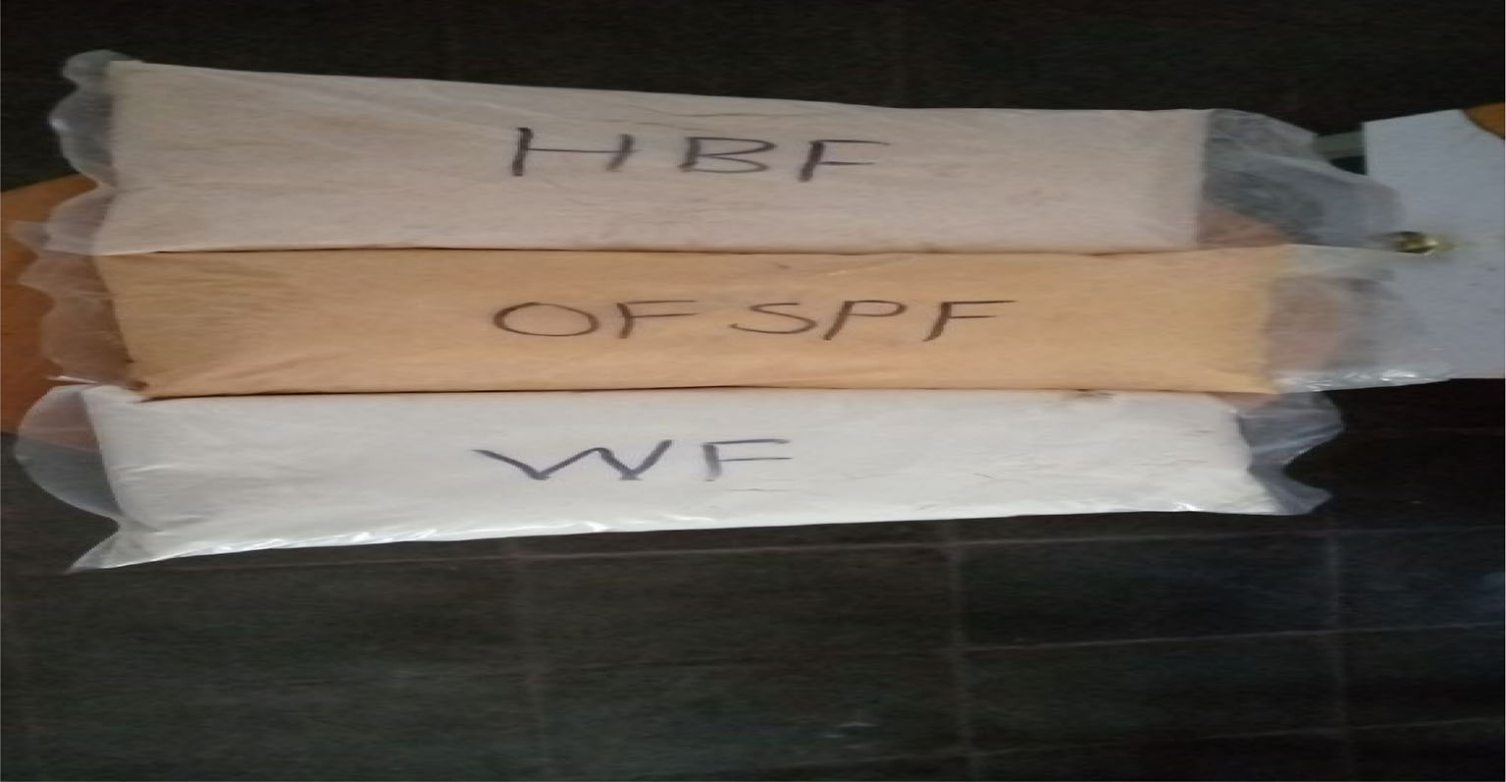
Flour of the raw materials.

The method described by Nshimiyimana ^24^ was used to prepare OFSP flour. The orange-fleshed sweet potato was peeled by hand after being sorted and washed with tap water. Peeled OFSP were sliced with a slicer machine (Model-CL 30, Robot @ Couple, Germany) and blanched in a water bath at 65°C for 10-minutes. The treated slices were drained and dried at 50°C in the oven for 24 hours. Then, haricot bean flour was prepared using the method of Kaur and Kapoor. ^25^ The seeds of the bean were sorted, washed, and soaked in distilled water in a water bath at 25°C for 24 hours at a ratio of 1:10 (w/v). The soaked seeds of the bean were washed twice in water, then rinsed with distilled water before being dried at 60°C in the oven for 48 hours. The dried OFSP flakes and dehulled beans were then milled into flour using a laboratory grinder (Model R 23, Robot @ Couple, Germany), and sieved with a 500 μm sieve size. Then the flour of each raw material was packed in a polyethylene plastic bag and held in a cool, dark place until further study ( Figure 1).

### 2.3 Development of OFSP and haricot bean flour enriched biscuits

**Figure 2.**
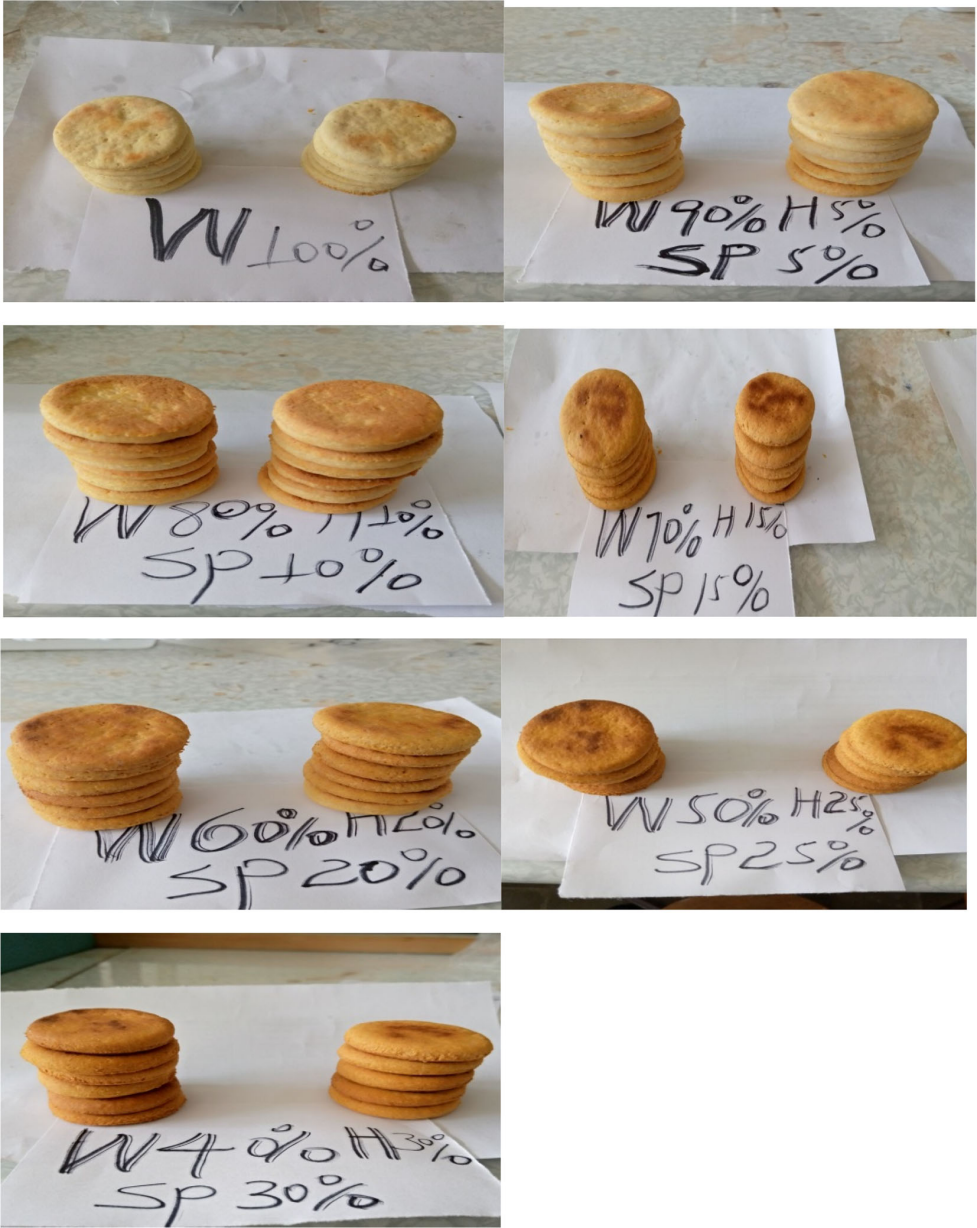
Diagrammatic pictures of biscuits prepared from blending of wheat, OFSP and Haricot bean with different ratios.

The formulation and development of biscuits are clearly presented in the current study. Accordingly, orange-fleshed sweet potato flour and haricot bean flour were blended into wheat flour at varying percentages of 5%, 10 %, 15%, 20%, 25%, and 30% ( Table 1 and Figure 2).

**Table 1.** Formulation of biscuits with orange-fleshed sweet potato and haricot bean flour.

Code	Blends	Ingredients				
		Baking powder(g)	Cooking oil(g)	Sugar (g)	Salt (g)	Water (ml)
P0	Control (100% wheat flour)	1.12	28	5	1	48
P1	W90%, H5%, OF5%	1.12	28	5	1	48
P2	W80%, H10%, OF10%	1.12	28	5	1	48
P3	W70%, H15%, OF15%	1.12	28	5	1	48
P4	W60%, H20%, OF20%	1.12	28	5	1	48
P5	W50%, H25%, OF25%	1.12	28	5	1	48
P6	W40%, H30%, OF30%	1.12	28	5	1	48

Where, W = Wheat, HBF = Haricot bean, OF = Orange fleshed sweet potato.

The biscuit dough was made following a commercial recipe and baking procedures. ^26^ Ingredients required to make biscuits were added in a similar amount to the different treatments in the experiment [baking powder (1.12 g/100 g), cooking oil (28 g/100 g), sugar (5 g/100 g), salt (1 g/100 g), and 48 ml of water] and mixed thoroughly ( Table 1). The biscuit dough was made by hand, and the total time spent mixing was 20-minutes. After the dough was prepared, it was manually sheeted to a thickness of 5 mm. The biscuits were then formed and cut to a diameter of 48 mm before being placed on a lightly greased baking tray. In a baking oven, the biscuits were baked for 12-minutes at a temperature of 200°C. The baking temperature was chosen based on some key evidence, such as the fact that carotene is susceptible to heat degradation and that a temperature of 200°C for 12-minutes exposure period results in better beta-carotene retention.

### 2.4 Chemical analysis

2.4.1 ***Functional properties of flours***

***Bulk density***

The bulk density of composite flour was determined using the method described by Oladele and Aina. ^27^ In a 50 ml measuring cylinder, 10 g of sample flour was placed. The cylinder was tapped repeatedly until the volume remained unchanged. The sample’s bulk density (g/ml) was determined by dividing the weight of the sample by the volume of the sample.

$$\text{Bulk density}(\frac{g}{\mathrm{ml}})=\frac{\text{weight of sample}\;(g)\;}{\text{volume of sample}\;(\mathrm{ml})}$$ (1)

***Dispersibility***

The method described by Kulkarni *et al.* ^28^ was used to assess dispersibility. Water was applied to each volume of 100 ml after 10 g of flour sample was weighed into a 100 ml measuring cylinder. The setup was vigorously stirred and left for three hours. The volume of settled particles was recorded and subtracted from 100. The difference was reported as percentage dispersibility.

% of dispersibility = 100−the volume of the settled particle (2)

***Water absorption capacity (WAC)***

The water absorption capacity of the flour samples was determined according to Aremu *et al.* ^29^ In a centrifuge tube, 1g of flour sample was mixed with 10 ml of distilled water and allowed to stand at room temperature for 30-minutes. The supernatant was collected in a 10 ml graduated cylinder after centrifugation at 5,000 rpm for 30 minutes. The amount of water absorbed per gram of flour sample was measured as ml of water absorbed per gram of flour sample.

$$\mathrm{WAC}\;\left(\mathrm{ml}\right)=\frac{w3-\left(w1+w2\right)}{w1}$$ (3)

Where w1 = weight of the sample, w2 = weight of the tube, w3 = weight of the sample after centrifugation.

***Oil absorption capacity (OAC)***

The oil absorption capacity of the flour samples was determined according to Aremu *et al.* ^29^ In a centrifuge tube, 1 g of flour sample was mixed with 10 ml of oil and allowed to sit at room temperature for 30-minute. It was centrifuged for 30 minutes at 5,000 rpm, with the supernatant collected in a 10 ml graduated cylinder. The amount of oil absorbed per gram of flour sample was measured as ml of oil absorbed per gram of flour sample.

$$\mathrm{OAC}\;\left(\mathrm{ml}\right)=\frac{w3-\left(w1+w2\right)}{w1}$$ (4)

Where w1 = weight of the sample, w2 = weight of the tube, w3 = weight of the sample after centrifugation.

2.4.2 ***Proximate and beta-carotene analyses of flour and biscuits***

The standard methods of the Association of Official Analytical Chemists ^30^ were used to assess proximate analyses. The Kjeldahl approach was used to calculate total nitrogen (TN). The crude protein content was measured by multiplying TN by a conversion factor of 6.25 (% protein = TN × 6.25), and the crude fat, crude fiber, ash content, and moisture content of the sample were determined according to the Association of Official Analytical Chemists. ^30^ The difference between 100 and (ash + protein + fiber + fat + moisture) was used to calculate the utilizable carbohydrate material. Using Atwater’s conversion factors, the energy content in kcal/100 g was calculated by multiplying the percentages of crude fat, crude protein, and carbohydrate by factors of 9, 4, and 4, respectively. Furthermore, the beta-carotene content of the sample was measured according to Muchoki *et al.* ^31^ In a 50 ml extraction conical centrifuge tube, 1 g of sample was weighed in duplicate and combined with 40 ml of acetone (High-Performance Liquid Chromatography grade). The samples were centrifuged for 60 seconds before being filtered through a Buchner funnel with suction. In a separating funnel, about 40 ml of petroleum ether was applied to the acetone extract. To prevent emulsion formation, distilled water was added slowly along the neck wall without shaking. Then, the two phases were separated, and the lower aqueous layer was discarded. To extract residual acetone, the sample was washed 3-4 times with distilled water (approximately 200 ml) each time. The upper layer was then collected into a 50 ml volumetric flask and residual water was removed using an anhydrous sodium sulfate filter arrangement. Using a UV-visible spectrophotometer, the absorbance of the ethereal extract was measured at 450 nm (Janeway, 96500, UK). The following formula was used to measure the concentration of β-carotene content.

$$\text{Beta carotene}\;\left(\mu \frac{g}{g}\right)=\frac{A*V*10,000}{A1*P}$$ (5)

Where A = Absorbance; V = Total extract volume (ml); P = Sample weight; A1 = 2592*β carotene extinction coefficient in petroleum ether.

2.4.3 ***Determination of physical properties of biscuit***

The spread factor (SF), diameter (D), and thickness (T) of the biscuit were calculated using different methods. ^32^ The diameters of the biscuits were determined by putting six biscuits horizontally edge-to-edge and rotating at a 90° angle for a duplicate measurement. The thickness of biscuits was determined by piling six biscuits on top of one another, then taking a duplicate reading by shuffling the biscuits. All of the measurements were performed in duplicates of six biscuits each, and the values per biscuit were calculated by dividing the total readings by six.

SF=D/T (6)

2.4.4 ***Sensory acceptability***

Panelists measured the sensory acceptability of biscuits based on their willingness to engage in the study. ^33^ With the help of the School of Nutrition, Food Science and Technology familiar assistance, around thirty consumer panelists were chosen randomly from food science students to test the sensory characteristics, such as appearance, taste, scent, crispiness, color, flavor, and overall acceptability, using a duplication experiment. A five-point hedonic scale was used (1 = very much hate, 2 = dislike, 3 = neither like nor dislike, 4 = like, and 5 = like very much).

2.4.5 ***Statistical data analysis***

The experimental data were subjected to one-way analysis of variance, and Duncan’s multiple range tests were used to detect the difference (p ≤ 0.05) between the mean values. Statistical analyses were performed with the statistical analysis system software package, version 9.0 standard methods (RRID: SCR_008567), and the data were presented as mean and standard deviation.

## 3. Results and Discussion

### 3.1 Functional properties of OFSP, wheat, and haricot bean flours

The bulk density, water absorption capacity, oil absorption capacity, and dispersibility of OFSP, wheat, and haricot bean flours are shown in Table 2. OFSP flour had a bulk density that was slightly higher than wheat and haricot bean flours. However, the water absorption capacity, oil absorption capacity, and dispersibility of the three flours were found to be significantly different from each other. Wheat flour had the highest, while OFSP flour had the lowest oil absorption capacity and dispersibility. Wheat flour had the lowest water absorption potential, while haricot bean flour had the highest.

**Table 2.** Functional properties of OFSP, wheat, and haricot bean flours.

Flour sample	BD (g/ml)	WAC (%)	OAC (%)	DESP (%)
WF	0.74 ± 0.03 ^b^	16.2 ± 0.56 ^c^	21.3 ± 0.14 ^a^	77.5 ± 2.12 ^a^
HBF	0.86 ± 0.01 ^b^	23.9 ± 0.98 ^a^	18.7 ± 0.14 ^b^	65.5 ± 0.70 ^b^
OFSPF	1.05 ± 0.06 ^a^	19.2 ± 0.70 ^b^	17.85 ± 0.35 ^c^	45.5 ± 2.12 ^c^

Where, BD = Bulk density, WAC = Water absorption capacity, OAC = Oil absorption capacity, DESP = Dispersibility, WF = Wheat flour, HBF = Haricot bean flour, OFSPF = Orange fleshed sweet potato flour. Values with the same column with different superscript letters are significantly different from each other (p < 0.05) and values are averages of duplicate readings (mean ± SD, n = 2).

In this analysis, the bulk density of OFSP (Alamura variety) flour is higher than that recorded by Tiruneh ^34^ for the Kulfo and Tulla varieties (0.74-0.62). The bulk density of haricot bean flour is higher than that of red kidney bean flour (0.51-0.55 g/ml), as stated by Khalil *et al*. ^35^ Wheat flour has a bulk density of 0.75 g/ml, which is almost identical to that stated by Biniyam. ^36^ This disparity may be due to varietal differences. The high bulk density of flour suggests that it is suitable for use in food preparations. However, the low bulk density of complementary foods would be advantageous. ^37^ Both haricot bean flour and OFSP flour have a higher bulk density than wheat flour, so adding haricot bean and OFSP flour to the composite flour would increase the bulk density of the composite flour as compared to wheat flour. The manufacture of confectioneries such as cakes, sweet pastries, doughnuts, and cookies benefits from an increase in flour bulk density. ^38^ This means that the flour’s heaviness and suitability for confectionery production are both positive. A rise in bulk density improves packaging performance. As a result, a larger amount may be packed into a smaller volume. ^39^

The water absorption capacity of haricot bean flour was higher than the value (17.3-16.8%) recorded by Shimelis *et al.* ^40^ on various haricot bean flour varieties. The WAC of the OFSP flour is lower than the values recorded by Tiruneh ^34^ for the Kulfo and Tulla varieties. The WAC of wheat flour is much higher than the value recorded by Biniyam ^36^ (8.5%). Many hydrophilic elements, such as carbohydrates and proteins (polar amino acid residues), have a strong affinity for water and contribute to the high WAC value. ^41^ Water absorption is reduced in flours with a lower proportion of polar amino acids and a higher proportion of nonpolar amino acids. ^42^ As a result, the observed differences in different flours may be due to differences in protein concentration, water interaction, and conformational characteristics. ^43^

Both haricot bean flour and OFSP flour have a higher WAC than wheat flour, so adding haricot bean and OFSP to the composite flour would improve the WAC of the composite flour compared with wheat flour. Increased water absorption weakens the dough and causes it to lose its development and stability. The ability to absorb water is essential for product consistency and bulking, as well as in baking applications. ^44^ Higher water absorption capacities, as suggested by Doescher *et al*. ^45^ and cited by Vieira *et al*., ^46^ may have contributed to the lower spread ratio. The oil absorption of wheat flour is higher than the values recorded by Baljeet *et al.* ^47^ and Suresh and Samsher ^38^ (16.9% and 14.6%, respectively). According to Shimelis *et al.* ^40^ the oil absorption capacity of haricot bean flour (18.7%) is lower than the values observed for various haricot bean varieties (24.9-35.2%). The oil absorption capacity of OFSP flour in this study is comparable to that reported by Tiruneh (16.8-18.4%) for Tulla and Kulfo varieties. ^34^

Due to the lipophilic quality of its constituents, wheat flour has a higher oil absorption capacity than OFSP flour and haricot bean flou. ^48^ Protein conformation, amino acid compositions, and surface polarity or hydrophobicity all play a role in lipophilicity. ^38^ Wheat flour has a higher OAC than OFSP and haricot bean flour, so adding haricot bean and OFSP to the composite flour would lower the OAC compared to wheat flour. When the OAC of composite flour is reduced, the taste and mouthfeel of the biscuits suffer. Dispersibility is a key metric for determining how well flour or flour blends will rehydrate with water without forming lumps. In this report, wheat flour had a substantially higher dispersibility than haricot bean and OFSP flour in this regard. The higher the dispersibility, the stronger the reconstitution property, and it’s what’s used to make a fine dough consistency during mixing. The property of dispersibility also defines flour’s propensity to detach from water molecules, exposing its hydrophobic behavior. Wheat flour has a higher dispersibility than OFSP and haricot bean flours, and the addition of haricot bean and OFSP will reduce the composite flour’s dispersibility as compared to wheat flour. Significantly lower dispersion of composite flour contributes to decreased dough consistency during mixing.

### 3.2 Proximate compositions and β-carotene content of OFSP, wheat, and haricot bean flours

Table 3 shows the proximate compositions and beta carotene of OFSP, wheat, and haricot bean flours. Moisture, protein, ash, fiber, and carbohydrate content were found to be substantially different between the three flours. Haricot bean flour has a slightly higher fat content than wheat and OFSP flour, which did not vary significantly. Wheat flour had the highest moisture content, while OFSP flour had the lowest. Haricot bean flour had the most protein, ash, fiber, and fat, while OFSP flour had the least protein and wheat flour had the least ash, fiber, and fat. The highest carbohydrate content was contained in OFSP flour, followed by wheat flour. Furthermore, the energy content of OFSP flour was substantially higher than that of haricot bean flour.

**Table 3.** Proximate compositions and β-carotene content of OFSP, wheat, and haricot bean flours (as the wet basis).

Flour	Moisture (%)	Crude Protein (%)	Crude Ash (%)	Crude Fiber (%)	Crude Fat (%)	CHO (%)	Energy (kcal/100 g)	β-carotene (μg/g)
WF	10.16 ± 0.07 ^a^	11.02 ± 0.60 ^b^	1.16 ± 0.08 ^c^	1.16 ± 0.07 ^c^	1.66 ± 0.32 ^b^	74.66 ± 1.07 ^b^	357.70 ± 1.64 ^ab^	ND
HBF	8.49 ± 0.23 ^b^	18.43 ± 1.38 ^a^	3.83 ± 0.70 ^a^	4.99 ± 0.20 ^a^	2.66 ± 0.14 ^a^	61.58 ± 2.38 ^c^	344.04 ± 7.27 ^b^	ND
OFSPF	4.49 ± 0.23 ^c^	5.25 ± 0.56 ^c^	2.16 ± 0.08 ^b^	4.16 ± 0.07 ^b^	1.33 ± 0.00 ^b^	82.51 ± 0.92 ^a^	363.04 ± 1.42 ^a^	126.64

Where, WF = Wheat flour, HBF = Haricot bean flour, OFSPF = Orange fleshed sweet potato flour, ND = Not detectable. Values with the same column with different superscript letters are significantly different from each other (p < 0.05) and values are averages of duplicate readings (mean ± SD, n = 2).

In terms of protein, ash (mineral), fiber, and fat content, haricot bean flour appears to have an advantage over wheat flour. The addition of OFSP flour appears to improve the fiber and ash content as well. According to Biniyam, ^36^ the product contains 13% moisture, 11% protein, 0.69% ash, 2% crude fiber, 2% fat, 71.3% carbohydrate, and 347.2 kcal/g energy. This research had higher moisture, crude fiber, and fat content than the current study and comparable protein content, but lower carbohydrate and energy content than the current study. Varietal disparities and measurement processes may be the reasons for these variations. For eight different haricot bean varieties grown in Ethiopia, researchers recorded 11.1-11.4% moisture, 18.0-22.1% protein, 2.9-4.3% ash, 4-5.9% crude fiber, 1.3-2.8% fat, 56.5-61.6% carbohydrate, and 330-343.2 kcal/g energy content. ^49^ Except for the higher moisture content of this sample, which may be attributed to drying methods or determination methods, all of the findings are consistent with the current study. For OFSP flour pretreated and dried with various methods, the results showed 4-8% moisture, 4-5.8% protein, 4.2-7.5% ash, 3.7-7.3% crude fiber, 0.9-2% fat, and 80-83.7% carbohydrate content. ^50^ Except for the lower ash content in this study, these findings are consistent with the current study. This inconsistency may be due to discrepancies in varietals, drying methods, and measurement methods. The addition of OFSP flour to biscuits appears to increase the overall β-carotene content. This study’s beta carotene finding is consistent with Takahata *et al.* ^51^ who found that the β-carotene content of sweet potato varieties varied between 0.1-266 g/g. The content of β-carotene in deep orange sweet potatoes ranged from 42.9-185.5 g/g. ^52^ In comparison to this analysis, other researchers recorded low values. Carotene levels in sweet potato varieties ranged from 16.8-18.5 g/g, according to Leighton. ^53^ The difference in OFSP is influenced by varieties, and there are high and low values of this nutrient. Variations in β-carotene content can be caused by variations in varieties, growing conditions, stages of maturity, harvesting and post-harvest handling, processing, and storage of OFSP. ^54^ ^,^ ^55^ Environmental influences, genetic factors, crop age, high irrigation levels, and cultivation management strategies may all have a major impact on a variety’s β-carotene content. ^56^

### 3.3 Effect of blending ratio of composite flour on proximate compositions and β-carotene content of biscuits

Table 4 shows the approximate composition and beta-carotene content of biscuits. The moisture, protein, ash, fiber, and fat content of the biscuit increased as the percentage of haricot bean flour and OFSP flour increased, as shown in Table 4. However, when 15% haricot bean flour and 15% OFSP flour are added to 100% wheat flour biscuits, the rise in proximate composition becomes most noticeable (control). However, when compared with the other proximate elements, carbohydrate content, and energy value showed the opposite trend. Both the carbohydrate content and energy value decreased as the proportion of wheat flour was reduced and the proportion of haricot bean and OFSP flour was increased. The addition of 15% haricot bean and 15% OFSP flour resulted in a significant decrease in carbohydrate content, whereas the addition of 25% haricot bean and 25% OFSP flour resulted in a significant decrease in energy value. Besides, wheat biscuits do not contain any β-carotene. As the proportion of haricot bean and OFSP flour increased, and β-carotene content in the composite flour biscuits increased significantly (p < 0.05).

**Table 4.** Effect of blending ratios on biscuits proximate compositions and β-carotene content on a wet weight basis (%).

Blends	Moisture (%)	Crude Protein (%)	Crude Ash (%)	Crude Fiber (%)	Crude Fat (%)	CHO (%)	Energy (kcal/100 g)	β-carotene (μg/g)
100%	6.99 ± 0.02 ^e^	11.69 ± 0.14 ^e^	1.99 ± 0.02 ^d^	1.49 ± 0.12 ^e^	1.71 ± 0.06 ^e^	76.14 ± 0.41 ^a^	366.53 ± 0.34 ^a^	-
W90%, H5%, OF5%	7.16 ± 0.07 ^e^	12.3 ± 0.43 ^e^	2.16 ± 0.11 ^cd^	1.83 ± 0.12 ^de^	1.74 ± 0.07 ^e^	74.8 ± 0.19 ^a^	364.10 ± 4.13 ^a^	29.61 ± 0.28 ^d^
W80%, H10%, OF10%	7.49 ± 0.04 ^de^	13.21 ± 0.27 ^de^	2.33 ± 0.02 ^c^	2.16 ± 0.45 ^cd^	1.87 ± 0.15 ^de^	72.93 ± 0.93 ^ab^	361.41 ± 5.55 ^ab^	30.67 ± 0.02 ^d^
W70%, H15%, OF15%	8.0 ± 0.15 cd	14.02 ± 0.25 ^cd^	2.66 ± 0.09 ^b^	2.5 ± 0.21 c	1.99 ± 0.04 ^cd^	70.82 ± 2.58 ^bc^	357.33 ± 1.18 ^ab^	37.28 ± 0.56 ^c^
W60%, H20%, OF20%	8.5 ± 0.42 c	15.22 ± 1.08 ^bc^	2.83 ± 0.03 ^b^	3.16 ± 0.05 ^b^	2.16 ± 0.11 ^bc^	68.12 ± 1.97 ^cd^	352.82 ± 0.48 ^ab^	61.16 ± 1.43 ^b^
W50%, H25%, OF25%	9.33 ± 0.45 ^b^	16.07 ± 0.90 ^ab^	3.16 ± 0.19 ^a^	3.66 ± 0.02 ^b^	2.33 ± 0.02 ^ab^	65.45 ± 0.72 ^d^	347.05 ± 12.36 ^bc^	63.08 ± 1.54 ^ab^
W40%, H30%, OF30%	10.96 ± 0.09 ^a^	17.15 ± 1.01 ^a^	3.33 ± 0.10 ^a^	4.33 ± 0.24 ^a^	2.49 ± 0.01 ^a^	61.73 ± 1.69 ^e^	337.93 ± 5.81 ^c^	64.43 ± 0.68 ^a^

Where, W = Wheat flour, H = Haricot bean flour, OF = Orange fleshed sweet potato flour. Values with the same column with different superscript letters are significantly different from each other (p < 0.05) and values are averages of duplicate readings (mean ± SD, n = 2).

Since OFSP and haricot bean flour have a higher water absorption ability and fiber content than wheat flour, the increase in moisture content in this study may be due to increasing the percentage of OFSP and haricot bean flour to wheat flour. This study confirmed the results of Biniyam, ^36^ who found that cookies made with wheat, quality protein maize, and carrot composite flour retained more moisture than cookies made with wheat flour. The latter two flours have higher fiber and water absorption abilities than wheat flour. According to Khaliduzzaman *et al.* ^57^ they replaced wheat flour with 20% potato flour while making biscuits, and the addition of potato flour increased the moisture content.

The results of this study agreed with those of Vieira, ^46^ who found that blending wheat flour with residue from king palm processing, which has a higher fiber content than wheat flour, results in a higher fiber content than wheat flour. According to Wang *et al.*, the hydroxyl group present in the fiber structure allows the higher total fiber in non-wheat flour to interact reasonably well with a large amount of water *.* ^58^ Since haricot bean flour has higher protein content than wheat flour, the rise in protein content in this study may be due to switching from haricot bean flour to wheat flour. The current study’s findings are consistent with those of Abayomi *et al.* ^59^ who recorded increased protein content in cookies made with sweet potato and fermented soybean flours, with the percentage of fermented soybean flour increasing the protein content of cookies. Cookies made with 30% soybean supplementation had a high protein content of 21.65%, while cookies made with 100% sweet potato flour had lower protein content.

According to Ndife *et al*. ^60^ the increased protein content in cookies from wheat and soya bean flours increased the percentage of soya bean flour, which increased the protein content of cookies by 8.75-24.65%. A similar finding was reported in a research study that showed an improvement in protein content in biscuit production from cassava-wheat-bambara flour blends with corresponding increases in the proportion of Bambara flour supplementation. ^61^ Since haricot bean and OFSP flour have higher ash content than wheat flour, the increase in the ash content of biscuits in this study may be due to increasing the percentage of haricot bean flour and the high dry matter content of OFSP flour to wheat flour.

The current study’s findings are consistent with those of Ndife *et al.* ^60^ who found that raising the percentage of soya bean flour in cookies increased the ash content of cookies by 2.15-2.95%, which is lower than the current study’s findings. Another study reported that cookies made from sweet potato and fermented soybean flour showed that raising the percentage of soya bean flour raised the ash content in cookies, with cookies made with 30% soybean supplementation having a high ash content of 2.57% and cookies made with 100% sweet potato flour having a lower ash content of 2.20%. ^59^ Similar findings were reported to increase the amount of ash content in cookies made from wheat and OFSP flour composite flours by increasing the percentage of OFSP flour in the composite flour by 1.05-1.17%, which is lower than the current research. ^62^ Because OFSP and haricot bean flour contain more fiber than wheat flour, the increase in fiber content in this study could be attributed to increasing the percentage of OFSP and haricot bean flour to wheat flour.

The result of the current study is consistent with Ndife *et al*. ^60^ who found that increased fiber content in cookies from wheat and soya bean flours increased the percentage of soya bean flour increased the fiber content of cookies by 3.29-5.73%. Biniyam ^36^ recorded higher fiber content in cookies made with wheat, quality protein maize, and carrot composite flours, the latter two having more fiber than wheat flour. The results of this study agree with those of Vieira *et al.* ^46^ who found that blending wheat flour with residue from king palm processing, which has a higher fiber content than wheat flour, results in a higher fiber content than wheat flour. Increased fiber content can help with waste passage by expanding the inside walls of the colon, making anti-constipation more efficient, lowering cholesterol levels in the blood, and lowering the risk of various cancers. ^63^

Since haricot bean flour has a higher fat content than wheat flour, the rise in crude fat content in this study may be attributed to increasing the ratio of haricot bean flour to wheat flour. The current results are consistent with those of Abayomi *et al.* ^59^ which showed an improvement in fat content in cookies made with sweet potato and fermented soybean flours while the amount of soya bean flour was increased. Cookies made with 30% soybean supplementation had a high-fat content of 5.25%, while cookies made with 100% sweet potato flour had a lower fat content of 1.22%. This finding was made in a research study by Biniyam ^36^ who found that cookies made with wheat, quality protein maize, and carrot composite flours had higher fat content than cookies made with wheat flour. The current study’s findings are consistent with Ndife *et al.* ^60^ reports of increased fat in cookies made with wheat and soya bean flours, where increasing the percentage of soya bean flour increased the fat content of cookies by 4.50-7.13%. Furthermore, Singh *et al.* ^64^ recorded a similar pattern in biscuit production from cassava-wheat-Bambara flour blends, with an increase in fat content and a corresponding increase in the proportion of Bambara flour supplementation.

The decrease in carbohydrate content of the cookie may be due to a rise in moisture, fat, ash, and fiber content as the proportion of OFSP and haricot bean flour in the formulation was increased, resulting in a decrease in carbohydrate content because carbohydrate is measured by difference. The current result was consistent with the results of Biniyam, ^36^ who found that cookies made from wheat, quality protein maize, and carrot composite flour had lower carbohydrate content and were higher in moisture, fat, ash, and fiber content. Furthermore, Vieira *et al.* ^46^ reported a reduction in the carbohydrate content of cookies by combining wheat flour with residue from king palm processing, which has a higher fiber, ash, and fat content than wheat flour. The energy content of the cookies follows the pattern of the carbohydrate content, as carbohydrate is the primary source of energy throughout the cookies. Based on Singh *et al.* ^64^ the recommended minimum daily energy requirement for an average Ethiopian man is 1820 kilocalories per person per day. Biscuits made in this study can provide about 18.56-20.13% of an average man’s daily energy requirements, with higher protein, and fiber content than wheat-based biscuits.

Consumption of β-carotene-rich foods, such as OFSP, which are also readily available in the home garden, can help to boost VA intake and alleviate VAD. ^65^ The increase in β-carotene content in the composite flour biscuits due to increasing the proportion of haricot bean and OFSP flour could be due to the OFSP flour. Thus, OFSP flour contains 126.64 g/g β-carotene, while wheat and haricot bean flour contain no β-carotene. According to the World Health Organization, the daily recommended dietary allowance of VA for pregnant/lactating women and children (6-59 months) is 800 μg and 400 μg, respectively. ^66^ Based on the United States Institute of Medicine, 1 retinol equivalence is equal to 12 μg β-carotene. ^67^ The results of the present study ranged from 2.47-5.37 μg/g VA retinol equivalence. Thus, the daily recommended dietary allowance of VA can be achieved by the consumption of 323.88 g biscuits (biscuits with the lowest OFSP), 148.98 g biscuits (biscuits with the highest OFSP) for pregnant/lactating women; and 161.94 g biscuits (biscuits with the lowest OFSP), and 74.49 g biscuits (biscuits with the highest OFSP) for children (6-59 months). This study's β-carotene content is significantly higher than that found by Laelago *et al*. ^62^ who discovered that increasing the proportion of OFSP flour in composite flour increased the beta-carotene content of biscuits made from wheat and OFSP flour by 0.55-13.11 g/g. According to Andualem *et al.* ^68^ biscuits made from OFSP and wheat flour contained 6.01 g/g and 2.86 g/g β-carotene, respectively, at baking temperatures of 200 and 220°C. Even though β-carotene content rises as OFSP content rises, the values vary from product to product. Since β-carotene is susceptible to heat degradation, the explanation may be due to varieties, growing conditions, stages of maturity, harvesting and post-harvest handling, processing, and storage of OFSP. ^55^ ^,^ ^56^ ^,^ ^69^

### 3.4 Effect of blending ratio of composite flour on physical characteristics of biscuits

Table 5 displays the physical characteristics of biscuits (diameter, thickness, and spread factor). The physical characteristics of biscuits did not improve significantly (p < 0.05) when haricot bean and OFSP flour were added. However, when compared with the control, the diameter, thickness, and spread factor decrease slightly when the proportion of haricot bean and OFSP flour is increased. The only exception was the diameter of biscuits produced with 40% wheat, 30% haricot bean, and 30% OFSP flour, which was slightly lower (p < 0.05) than the control (100% wheat flour).

**Table 5.** Effect of blending ratios on physical characteristics of biscuits.

Blends	Diameter (cm)	Thickness (cm)	Spread factor
W100%	4.87 ± 0.07 ^a^	0.51 ± 0.77 ^a^	9.56 ± 1.29 ^a^
W90%, H5%, OF5%	4.77 ± 0.03 ^ab^	0.50 ± 0.77 ^ab^	9.55 ± 1.38 ^a^
W80%, H10%, OF10%	4.65 ± 0.07 ^ab^	0.49 ± 0.77 ^a^	9.50 ± 1.33 ^a^
W70%, H15%, OF15%	4.54 ± 0.21 ^ab^	0.48 ± 0.77 ^a^	9.45 ± 1.06 ^a^
W60%, H20%, OF20%	4.44 ± 0.36 ^ab^	0.47 ± 0.77 ^a^	9.41 ± 0.78 ^a^
W50%, H25%, OF25%	4.32 ± 0.45 ^ab^	0.46 ± 0.77 ^a^	9.34 ± 0.57 ^a^
W40%, H30%, OF30%	4.14 ± 0.34 ^b^	0.45 ± 0.70 ^a^	9.26 ± 0.68 ^a^

Where, W = wheat flour, H = Haricot bean flour, OF = orange fleshed sweet potato flour. Values with the same column with different superscript letters are significantly different from each other (p < 0.05) and values are averages of duplicate readings (mean ± SD, n = 2).

With the addition of OFSP, different studies recorded a decrease in the diameter, ^62^ ^,^ ^64^ thicknesses, ^64^ ^,^ ^70^ ^,^ ^71^ and spread factor ^62^ ^,^ ^72^ of wheat cookies. These findings are consistent with the fact that the addition of haricot bean and OFSP flour reduced the diameter, thickness, and spread ratio of biscuits in the current report. The addition of haricot bean flour in addition to the OFSP flour may have resulted in a small decrease in the diameter, thickness, and spread ratio of the biscuits in this report. The haricot bean’s higher protein content may have some gluten-substituting properties. The haricot bean’s higher dispersibility than OFSP may have a secondary effect of lowering the spread ratio. Rapid partitioning of free water to hydrophilic sites during mixing increased dough viscosity, restricting biscuit spread. ^73^ As a result of the addition of OFSP and haricot bean flour to cookies, the distribution of the cookies is reduced.

### 3.5 Effect of blending ratio of composite flour on sensory acceptability of biscuits

Table 6 demonstrates the sensory acceptability of biscuits (color, shape, flavor, taste, texture, crispiness, and overall acceptability). Except for the one with the highest haricot bean and OFSP flour (W40%, H30%, and OF30%), the appearance of the composite flour biscuits did not display a substantial difference (p < 0.05) compared with the 100% wheat flour biscuits (control). The color of all the composite biscuits did not vary significantly from the control (p < 0.05). The crispiness of composite flour biscuits containing 20, 25, and 30% haricot bean flour and OFSP flour was slightly lower (p < 0.05) than that of biscuits made entirely of wheat. All of the composite flour biscuits, except W60%, H20%, OF20%, and W40%, H30%, OF30%, tasted and were accepted in the same way as the 100% wheat flour cookies in terms of flavor, taste, and overall acceptability.

**Table 6.** Effect of blending ratios on sensory properties of biscuits.

Blends	Appearance	Color	Flavor	Crispiness	Taste	OAA
W100%	4.11 ± 0.80 ^ab^	4.08 ± 0.84 ^a^	3.73 ± 1.05 ^b^	4.26 ± 1.05 ^a^	3.85 ± 1.11 ^b^	3.98 ± 0.65 ^b^
W90%, H 5%, OF5%	4.21 ± 0.76 ^ab^	4.10 ± 0.75 ^a^	3.88 ± 0.84 ^ab^	4.25 ± 0.85 ^a^	3.91 ± 0.92 ^b^	4.00 ± 0.68 ^ab^
W80%, H10%, OF10%	4.28 ± 0.55 ^a^	4.16 ± 0.78 ^a^	3.90 ± 0.75 ^ab^	4.16 ± 1.06 ^a^	3.95 ± 0.81 ^ab^	4.05 ± 0.74 ^ab^
W70%, H15%, OF15%	4.30 ± 0.80 ^a^	4.18 ± 0.74 ^a^	3.98 ± 0.91 ^ab^	4.06 ± 1.00 ^ab^	3.98 ± 0.96 ^ab^	4.13 ± 0.59 ^ab^
W60%, H20%, OF20%	4.36 ± 0.75 ^a^	4.23 ± 0.78 ^a^	4.10 ± 0.93 ^a^	3.70 ± 1.09 ^bc^	4.25 ± 0.77 ^a^	4.25 ± 0.75 ^a^
W50%, H25%, OF25%	3.93 ± 0.91 ^a^	3.96 ± 0.95 ^a^	3.71 ± 0.86 ^b^	3.60 ± 1.09 ^c^	3.75 ± 1.05 ^bc^	3.91 ± 0.92 ^b^
W40%, H30%, OF30%	3.41 ± 1.10 ^c^	3.51 ± 1.12 ^a^	3.21 ± 0.94 ^c^	3.51 ± 1.09 ^c^	3.43 ± 0.76 ^c^	3.50 ± 0.79 ^c^

Where, W = Wheat flour, H = Haricot bean flour, OF = Orange fleshed sweet potato flour, OAA = Overall acceptability Values with the same column with different superscript letters are significantly different from each other (p < 0.05) and values are averages of duplicate readings (mean ± SD, n = 2).

In comparison with 100% wheat flour biscuits, the appearance, color, flavor, taste, and overall acceptability scores of the biscuits improved with the addition of haricot bean and OFSP flour up to H20%, OF20%. The crispiness of the biscuits, on the other hand, decreased as haricot bean and OFSP flour were added. Despite this, the crispiness of the composite flour biscuits is not substantially different from the control up to the addition of 15% haricot and 15% OFSP. The most critical quality attributes that can affect the acceptability of a food product and a consumer’s buying decision are its appearance and color. ^74^ The current study’s appearance results are consistent with the findings of, ^68^ which found that biscuits made from composite flours containing 70% wheat and 30% OFSP flour had a higher appearance value. The attractive color of OFSP may have contributed to the composite flour biscuits’ increased color acceptability. ^75^ The color acceptability of the current study agrees with the results of, ^57^ who found that biscuits made with 70% wheat and 30% OFSP composite flour had higher color acceptability. Similarly, ^75^ discovered that the color acceptability pattern of biscuits made from composite wheat and OFSP flour increased over time. The addition of haricot bean and OFSP flour increased the taste acceptability score of the cookies, which may be attributed to the sweetness of the OFSP flour.

The current study’s taste results are consistent with the findings of Afework *et al.* ^68^ who recorded a higher taste acceptability score for biscuits made with 70% wheat and 30% OFSP composite flours. According to Onabanjo and Ighere, the taste acceptability score of biscuits made from wheat and OFSP composite flours improved in the same way. ^75^ A food product’s flavor is a mixture of taste and aroma, as well as other sensory qualities. The improvement in the flavor of the cookies with increased haricot bean and OFSP may be attributed to the sweetness of the OFSP, similar to the taste attribute. According to Terefe, the same trend of increasing flavor acceptability scores was observed in flatbread made from wheat and OFSP composite flours. ^76^ Crispiness is one of the most important textural characteristics of dry snack foods, indicating freshness and high quality. A crisp product should, in general, be strong and snap easily when bent, emitting a crunchy sound. ^77^ The decrease in biscuit crispiness as the proportion of haricot bean flour and OFSP flour is increased may be attributed to the increased moisture content of the biscuits as the proportion of haricot bean and OFSP flour is increased. The decrease in biscuit crispiness as the proportion of haricot bean and OFSP flour is increased may be attributed to the increased moisture content of the biscuits as the proportion of haricot bean and OFSP flour is increased. According to Manley, ^78^ a biscuit’s structure will not be crisp at higher moisture levels. Finally, overall acceptability is a metric that assesses a product’s overall acceptance. Except for crispiness, the overall acceptability of the biscuits improved as the haricot bean and OFSP flour content increased. With the addition of OFSP flour to wheat flour, Onabanjo and Ighere ^75^ recorded an improvement in overall biscuit acceptability. In the end, all of the composite flour biscuits, except for the W40%, H30%, and OF30% were as good as or better than the 100% wheat biscuits in terms of acceptability.

## 4. Conclusion

Based on the findings of the current study, blending wheat with OFSP and haricot bean during biscuit formulation appears to be promising in improving nutritional quality, sensory acceptability, and physical and chemical properties. Biscuits from wheat, haricot bean, and OFSP composite flours had increased protein content where their compositions could alleviate protein-energy malnutrition. The inclusion of OFSP in the composite biscuit also significantly increased the amount of beta carotene. It is anticipated that these foodstuffs can lessen food insecurity and VAD. Furthermore, the composite flour biscuits had better sensory acceptability than biscuits produced from 100% wheat.

## Data availability

OSF: Underlying data for “Effect of blending ratio of wheat, orange fleshed sweet potato and haricot bean flour on proximate compositions, β-carotene, physicochemical properties and sensory acceptability of biscuits”; https://doi.org/ 10.17605/OSF.IO/Y3ANX. ^79^

Data is available under the terms of the Creative Commons Attribution 4.0 International license (CC-BY 4.0).

## Ethical consideration

The study was reviewed and approved by the Institutional Review Board of the College of Medicine and Health Sciences of Hawassa University. Written permission was also obtained from the School of Nutrition, Food Science and Technology. Informed written consent was obtained from the panelists before the actual sensory data was collected. The purposes and importance of this study were explained to all panelists. The responses of each panelist were kept confidential by coding. The data was collected and analyzed anonymously.
