# Supplementary material for: Effect of blending ratio of wheat, orange fleshed sweet potato and haricot bean flour on proximate compositions, β-carotene, physicochemical properties and sensory acceptability of biscuits’
Source: F1000Res. 2022 Mar 11;10:506. Originally published 2021 Jun 28. [Version 2] doi: 10.12688/f1000research.52634.2 (PMC8961195; doi:10.12688/f1000research.52634.2)
Supplement: Peer review history

# Peer review: Reviewer response for version 2

**DOI:** 10.5256/f1000research.119918.r127133

**Published:** 2022-03-28

**Authors:** Elisa Julianti

**Affiliations:** 1 Food Science Department, Faculty of Agriculture, Universitas Sumatera Utara, Medan, Indonesia; 2 Centre for Tubers and Roots Crop Study, Universitas Sumatera Utara, Medan, Indonesia

The author has made revisions in accordance with the input and recommendations given previously so that this paper can be indexed.

Is the work clearly and accurately presented and does it cite the current literature?

Partly

If applicable, is the statistical analysis and its interpretation appropriate?

Yes

Are all the source data underlying the results available to ensure full reproducibility?

Partly

Is the study design appropriate and is the work technically sound?

Yes

Are the conclusions drawn adequately supported by the results?

Yes

Are sufficient details of methods and analysis provided to allow replication by others?

Partly

Reviewer Expertise:

Food process technology

I confirm that I have read this submission and believe that I have an appropriate level of expertise to confirm that it is of an acceptable scientific standard.

# Peer review: Reviewer response for version 1

**DOI:** 10.5256/f1000research.55936.r91242

**Published:** 2021-09-06

**Authors:** Mesfin Wogayehu Tenagashaw

**Affiliations:** 1 Faculty of Chemical and Food Engineering, Bahir Dar Institute of Technology, Bahir Dar University, Bahir Dar, Ethiopia

In general, the manuscript does fit consideration for indexing, provided that some improvement is made by the authors based on comments/suggestions given by reviewers. It requires both technical and mainly typographical editorials/corrections. I have tried to go through it and thus made the following points:

**Title**

- The title is too long and also it needs some modification; I suggest the following: "Effect of blending ratio on the physicochemical properties and sensory acceptability of biscuits prepared from blends of wheat-orange-fleshed sweet potato-haricot bean flours".

**Abstract**

- The background information mentioned only protein-energy malnutrition as the main problem; what about the micronutrient problem prevailing in the country? For example, the conclusion mentioned vitamin A deficiency.

**General**

- There are a number of grammatical errors (typographical issues) that need to be edited/corrected throughout the manuscript.
- There are lots of missing references (citations) almost in all sections. That should be revised/corrected. 

**Introduction**

- The Introduction section needs revisions; it should specifically focus on issues of malnutrition in Ethiopia and also its coherence/logical flow needs revisiting.
- The rationale of the work is not clearly indicated. As to me, the rationale can still be made stronger by adding other ideas such as the contribution of OSFP to fight vitamin A deficiency. 

**Methods**

- The Methods section also needs a thorough revision: repetition of ideas and also re-ordering of the sub-sections and editorial things on these sections is required.
- It is not clear from where the OFSP was collected.
- Sections 2.2 and 2.4 should be merged as they refer to the same thing.

**Results and Discussion**

- The Results and Discussion section is relatively well written. However, tables (Results) should normally appear immediately after the first mention; this is not the case in this manuscript. Kindly edit accordingly.
- Values of both results of the research and those of cited reports (for comparison purposes) are not shown. This should be made so as to make things clear to the reader and also improve the quality of the article.
- Statistical significance has not been shown in most of the reports of the results. Only mere reports were made. To my knowledge, this should be corrected; otherwise, the possibility of indexing would be reduced.

**Conclusion**

- In the conclusion section, the phrase ".....with more OFSP supplementation....." is not clear. In the first place, the work was not supplementation.

Is the work clearly and accurately presented and does it cite the current literature?

Partly

If applicable, is the statistical analysis and its interpretation appropriate?

Partly

Are all the source data underlying the results available to ensure full reproducibility?

Yes

Is the study design appropriate and is the work technically sound?

Yes

Are the conclusions drawn adequately supported by the results?

Partly

Are sufficient details of methods and analysis provided to allow replication by others?

Partly

Reviewer Expertise:

Complementary food processing, food quality and safety

I confirm that I have read this submission and believe that I have an appropriate level of expertise to confirm that it is of an acceptable scientific standard, however I have significant reservations, as outlined above.

# Response

**Published:** 2022-02-25

**Authors:** Gesessew L. Kibr

**Affiliations:** Shambu Campus, Wollega University, Ethiopia

The title has been modified to make it shorter and more precise. The background information and abstract conclusion are narrated while taking into account both protein energy malnutrition and vitamin A deficiency. The contribution of OFSP clearly indicates the logical flow of the idea concerning malnutrition and the rationality of the problem. Nutritional and food science perspectives are also discussed. OFSP's origin is stated in the study setting. The merging of related sub-headings and re-ordering of subsections were done carefully. The enhancement to increase the impressiveness of the result has been completed. Only statistically significant values were indicated in the discussion section. A concerted effort was made to ensure that the comparison point was both impressive and adequate. To bring the study's work to a close, paraphrasing was used in the conclusion section. Grammatical errors, referencing, and citation correction were all considered. Order and coherence were also maintained adequately throughout the document.

# Peer review: Reviewer response for version 1

**DOI:** 10.5256/f1000research.55936.r91530

**Published:** 2021-08-24

**Authors:** Elisa Julianti

**Affiliations:** 1 Food Science Department, Faculty of Agriculture, Universitas Sumatera Utara, Medan, Indonesia; 2 Centre for Tubers and Roots Crop Study, Universitas Sumatera Utara, Medan, Indonesia

The paper investigates the proximate composition, physical properties and sensory acceptability of biscuits made from wheat, orange-fleshed sweet potato (OFSP) powder and haricot bean flour. The authors explain the societal and economic value by substituting imported wheat flour with domestic crops in the production of food products such as biscuits. The content of the paper is in line with the scope of the journal and addresses an important research question. No doubt about that. However, in my point of view, the paper does need some revision before indexing. Please find my specific comments below:

- The authors used different terms for OFSP and haricot bean (powder for OFSP and flour for haricot bean), please make a justification about these terms.
- In the introduction, the authors state that from the results of the study it is expected that a good blend of wheat, OFSP, and haricot bean flours will produce nutritionally enriched biscuits, while in the methodology, the observations are only made on the proximate composition of biscuits. No observations on micronutrients.
- There was no observation of the color and texture of the biscuit. The addition of OFSP and haricot bean flour must have a great effect on these two parameters. Observation of these two parameters would highly improve the credibility of the manuscript. This can also justify the assessment results from the panelists because the consumer acceptance test is only carried out by 30 panelists.
- In Table 3, the unit of energy should be kcal/100g.
- Does it cite the current literature? Partly, as only about 33% of the references are within the last 10 years.

Is the work clearly and accurately presented and does it cite the current literature?

Partly

If applicable, is the statistical analysis and its interpretation appropriate?

Yes

Are all the source data underlying the results available to ensure full reproducibility?

Partly

Is the study design appropriate and is the work technically sound?

Yes

Are the conclusions drawn adequately supported by the results?

Yes

Are sufficient details of methods and analysis provided to allow replication by others?

Partly

Reviewer Expertise:

Food process technology

I confirm that I have read this submission and believe that I have an appropriate level of expertise to confirm that it is of an acceptable scientific standard, however I have significant reservations, as outlined above.

# Response

**Published:** 2022-02-25

**Authors:** Gesessew L. Kibr

**Affiliations:** Shambu Campus, Wollega University, Ethiopia

The consistency of OFSP and haricot bean (powder for OFSP and flour for haricot bean) is taken into account. Methodological observations on beta carotene content are combined. The sensory test was carried out in accordance with the guidelines of Hawassa University's school of nutrition, food science, and technology, with a panel of 30 panelists. Texture was never intended to be a study variable from the start. Furthermore, a more detailed explanation of the color is being considered. In Table 3, Kcal/100g is used as a correction. To update the manuscript, recently published papers are reviewed and comparisons was done accordingly.

# Peer review: Reviewer response for version 1

**DOI:** 10.5256/f1000research.55936.r88506

**Published:** 2021-08-05

**Authors:** Warinporn Klunklin

**Affiliations:** 1 School of Agro-Industry, Faculty of Agro-Industry, Chiang Mai University, Chiang Mai, Thailand

This manuscript studied the blending ratio of different types of flour substituted in wheat flour for biscuit preparation.

 The recommendations are listed below. 

- The information in the Introduction section was explained in the nutrition field while the Methods were not determined by any nutrition factors after changing the ingredients of the fortified biscuits.
- This manuscript was more like in the food science field. The authors need to rewrite the whole Introduction by adding some new studies of biscuit production, the gap of recent works, and the use of beans and pulses in the food industry. 
- The biscuit preparation can be easily understood by adding all recipes to a separate table. 
- The sensory test was inappropriate to interpret the results due to the use of a small group of panellists that were involved with this study (only 30 people). Normally, the sensory test was acceptable at 100 untrained panellists. The researchers also used only a 5-point hedonic scale which is too rough. It is quite hard to believe the results from this part. 
- The results were unclear and must be improved. It is better to take a photo of OFSF and haricot bean flour and also the composited flour. I do believe that the colour of biscuits should interfere with orange sweet potato flour colour. Moreover, varying the composition of flour in the biscuit formula normally changes the texture of biscuits affecting consumers liking scores. This parameter should be evaluated. That's why this manuscript can't ensure reproducibility.
- The Conclusion was inadequate to summarise the whole work.

Is the work clearly and accurately presented and does it cite the current literature?

Partly

If applicable, is the statistical analysis and its interpretation appropriate?

Yes

Are all the source data underlying the results available to ensure full reproducibility?

Partly

Is the study design appropriate and is the work technically sound?

Yes

Are the conclusions drawn adequately supported by the results?

Partly

Are sufficient details of methods and analysis provided to allow replication by others?

Partly

Reviewer Expertise:

Development of biscuits, utilisation of agricultural waste, chitosan extraction

I confirm that I have read this submission and believe that I have an appropriate level of expertise to confirm that it is of an acceptable scientific standard, however I have significant reservations, as outlined above.

# Response

**Published:** 2022-02-24

**Authors:** Gesessew L. Kibr

**Affiliations:** Shambu Campus, Wollega University, Ethiopia

The information in the introduction section describes the nutritional concepts and their application from an ingredients' perspective in the methodology part as well. Additionally, nutritional and food science perspectives are discussed, with an emphasis on biscuit production, recent discrepancies, and the use of beans and pulses in the food industry. A separate table is used for presenting the recipes. The manuscript includes photographs of the raw materials and blended biscuits. The sensory test was carried out in accordance with the guidelines of Hawassa University's school of nutrition, food science, and technology, with a panel of 30 panelists. Texture was never intended to be a study variable from the start. Corrections were made as much as possible in the conclusion section to summarize the work of the study.
